# The *Arabidopsis thaliana* E3 Ubiquitin Ligase BRIZ Functions in Abscisic Acid Response

**DOI:** 10.3389/fpls.2021.641849

**Published:** 2021-03-16

**Authors:** Katrina J. Linden, Mon Mandy Hsia, Yi-Tze Chen, Judy Callis

**Affiliations:** ^1^Department of Molecular and Cellular Biology, University of California, Davis, Davis, CA, United States; ^2^Integrated Genetics and Genomics Graduate Program, University of California, Davis, Davis, CA, United States; ^3^Biochemistry and Molecular Biology Graduate Program, University of California, Davis, Davis, CA, United States; ^4^Plant Biology Graduate Program, University of California, Davis, Davis, CA, United States

**Keywords:** *Arabidopsis*, E3 ligase, abscisic acid, ubiquitin, hormone signaling, germination, ABI5, ABA2

## Abstract

The ubiquitin system is essential for multiple hormone signaling pathways in plants. Here, we show that the *Arabidopsis thaliana* E3 ligase BRIZ, a heteromeric ligase that consists minimally of BRIZ1 and BRIZ2 proteins, functions in abscisic acid (ABA) signaling or response. *briz1* and *briz2* homozygous mutants either fail to germinate or emerge later than wild-type seedlings, with little cotyledon expansion or root elongation and no visible greening. Viability staining indicates that *briz1* and *briz2* embryos are alive but growth-arrested. Germination of *briz* mutants is improved by addition of the carotenoid biosynthetic inhibitor fluridone or gibberellic acid (GA_3_), and *briz* mutants have improved development in backgrounds deficient in ABA synthesis (*gin1-3/aba2*) or signaling (*abi5-7*). Endogenous ABA is not higher in *briz2* seeds compared to wild-type seeds, and exogenous ABA does not affect *BRIZ* mRNAs in imbibed seeds. These results indicate that *briz* embryos are hypersensitive to ABA and that under normal growth conditions, BRIZ acts to suppress ABA signaling or response. ABA signaling and sugar signaling are linked, and we found that *briz1* and *briz2* mutants excised from seed coats are hypersensitive to sucrose. Although *briz* single mutants do not grow to maturity, we were able to generate mature *briz2-3 abi5-7* double mutant plants that produced seeds. These seeds are more sensitive to exogenous sugar and are larger than seeds from sibling *abi5-7 BRIZ2/briz2-3* plants, suggesting that BRIZ has a parental effect on seed development. From these data, we propose a model in which the BRIZ E3 ligase suppresses ABA responses during seed maturation and germination and early seedling establishment.

## Introduction

The ubiquitin system is a post-translational protein modification system in which E1, E2, and E3 enzymes catalyze the attachment of one or more ubiquitins to substrate proteins. E3s, or ubiquitin ligases, facilitate the transfer of activated ubiquitin from an E2 to the substrate, either directly or by forming a thioester bond with the ubiquitin prior to its transfer to the substrate. E3s are the key specificity components in ubiquitination of substrate proteins and therefore their presence and activity are important points of regulation. Additional proteins in the ubiquitin system modulate the activity, localization, or abundance of ubiquitinated proteins (Oh et al., 2018), remove ubiquitin, or modulate the above processes. In plants, the ubiquitin system affects multiple developmental and environmental responses and hormone signaling pathways (reviewed in Vierstra, 2011; Sadanandom et al., 2012; Gibbs et al., 2014; Kelley, 2018; Miricescu et al., 2018), including the abscisic acid (ABA) biosynthetic and signaling pathways (reviewed in Liu and Stone, 2011; Yu et al., 2016; Jurkiewicz and Batoko, 2018; Zhang et al., 2019).

Abscisic acid is a hormone that affects many aspects of plant development and stress responses (reviewed in Finkelstein, 2013; Yoshida et al., 2019). It influences embryo development, seed maturation, dormancy, germination, growth, senescence, and allows plants to respond appropriately to drought, salinity, and pathogens. The ABA biosynthetic gene *GLUCOSE INSENSITIVE 1* (*GIN1*, also called *ABA DEFICIENT 2*, *ABA2*) encodes a short-chain dehydrogenase/reductase enzyme that converts xanthoxin to ABA aldehyde, and mutants have reduced ABA levels (Cheng et al., 2002; González-Guzmán et al., 2002). The canonical ABA signaling pathway (reviewed in Cutler et al., 2010) begins with ABA perception by PYRABACTIN/PYRABACTIN-LIKE (PYR/PYL) proteins, followed by inhibition of clade A type 2C protein phosphatases (PP2Cs). Sucrose non-fermenting related-1 (SnRK) 2 type kinases previously held in check by PP2Cs are subsequently activated and can phosphorylate downstream targets including bZIP transcription factors such as ABI5 (Zhang et al., 2019) or ion channels in guard cells (Munemasa et al., 2015). The Raf-like kinase RAF10 interacts with PP2Cs and phosphorylates SnRK2s and several downstream transcription factors (Nguyen et al., 2019), and multiple other Raf-like kinases are important for SnRK2 phosphorylation and ABA responses (Lin et al., 2020). Additionally, the glycogen kinase-like protein BRASSINOSTERIOD INSENSITIVE 2 (BIN2) phosphorylates PP2Cs (Cai et al., 2014), suggesting that Raf-like and BIN2 kinases modulate the core ABA signaling pathway.

Abscisic acid signaling is also regulated by the ubiquitin system, and dozens of E3 ligases involved have been identified (reviewed in Yu et al., 2016). The substrate binding subunit of a CULLIN4-type E3 ligase, DDA1 (for DET1-, DDB1-ASSOCIATED1), interacts with PYLs 4, 8, and 9 *in vivo* and facilitates the proteasomal degradation of PYL8 (Irigoyen et al., 2014). RING FINGER OF SEED LONGEVITY (RSL1) is a plasma membrane E3 that regulates intracellular trafficking of PYL4 (Bueso et al., 2014). The E3s PUB12 and PUB13 target the PP2C ABI1 for degradation (Kong et al., 2015), and the E3s RGLG1 and RGLG5 ubiquitinate the PP2Cs PP2CA, ABI2, and HAB2 *in vitro* and regulate their abundance *in vivo* (Wu et al., 2016). PHLOEM PROTEIN 2-B11 (PP2-B11) is an F-box protein in an SCF-type E3 that targets the kinase SnRK2.3 for degradation (Cheng et al., 2017).

Components downstream of SnRK2 kinases are also modulated by the ubiquitin system. The E3 RHA2b ubiquitinates MYB30, a transcription factor that negatively regulates ABA signaling, *in vitro*, interacts with it *in vivo*, and affects its accumulation in plants (Zheng et al., 2018). The E3 AIP2 (ABI3-interacting protein) ubiquitinates the transcription factor ABI3 *in vitro* and reduces its levels *in vivo* (Zhang et al., 2005). Loss of the E3 DESPIERTO results in decreased *ABI3* and *ABI4* expression during seed development, reduced ABA sensitivity during germination, and loss of dormancy (Barrero et al., 2010). LOSS OF GDU2 (LOG2, also referred to as AIRP3) binds to and monoubiquitinates the mature form of RD21, a drought-induced cysteine protease, *in vitro*, and *LOG2* loss-of-function mutants are ABA hyposensitive (Kim and Kim, 2013).

Many E3s that affect ABA signaling do not have identified substrates to date. Reduced expression of the E3 RING-H2 FINGER A (RHA2a) or over-expression with the 35S promoter result in diminished or enhanced ABA responses, respectively (Bu et al., 2009). TUBBY9 encodes an F-box protein, a substrate-specificity subunit of the CUL1-based E3 ligases, and loss-of-function mutants have reduced ABA sensitivity, while TUBBY9 over-expression results in ABA hypersensitivity (Lai et al., 2004). RING DOMAIN AND DOMAIN-OF-UNKNOWN-FUNCTION 1 and 2 (RDUF1 and RDUF2) loss-of-function mutants are ABA-hyposensitive (Kim et al., 2012).

ABI5 is a bZIP transcription factor with a major role in seed germination (Lopez-Molina et al., 2001, and reviewed in Skubacz et al., 2016). The first loss-of-function allele, *abi5-1*, was recovered in a screen for ABA-resistant germination in *Arabidopsis thaliana* (Finkelstein, 1994). Characterization of multiple loss-of-function *abi5* alleles indicates that plants lacking *ABI5* are not phenotypically different from wild-type plants in the absence of exogenous ABA, including in their stomatal responses under low water potential conditions (Finkelstein, 1994; Nambara et al., 2002). ABI5 protein is highest in dry seeds (Piskurewicz et al., 2008) and its abundance is modulated by multiple E3 ligases. *KEEP ON GOING* (*KEG*) encodes an E3 whose loss-of-function mutants accumulate ABI5 protein and are hypersensitive to ABA (Stone et al., 2006). Recombinant KEG ubiquitinates ABI5 *in vitro* (Liu and Stone, 2010). The E3 CUL4-based substrate specificity factors DWA1 and 2 (DWD hypersensitive to ABA1 and 2) function in ABA signaling and negatively affect ABI5 levels *in vivo* (Lee et al., 2010a). HYPERSENSITIVE DCAF 1 (ABD1) is another substrate receptor of a CUL4-based ligase that interacts with ABI5 and plays a role in ABI5 degradation (Seo et al., 2014).

Loss-of-function mutants of many of the E3s described above have phenotypes that are modest or only visible under exogenous ABA treatment, with the exception of *keg* mutants, which grow slowly and have arrested growth after developing one set of true leaves (Stone et al., 2006). In addition to ABI5, *KEG* is implicated in degradation of other bZIP transcription factors (Chen et al., 2013) and is involved in intracellular trafficking and pathogen responses (Gu and Innes, 2012). These additional functions likely contribute to the *keg* loss-of-function phenotype.

Previously, we identified T-DNA insertion mutations in genes encoding two related E3 proteins called BRIZ1 (for BRAP2-RING-Znf Domain) and BRIZ2 (Hsia and Callis, 2010). Homozygous T-DNA insertion lines of either *BRIZ* gene have the same severe phenotype of post-germination growth arrest, suggesting that both proteins are required for the same processes. We reported that BRIZ1 and BRIZ2 proteins preferentially form heteromers *in vitro* and are found in the same complex *in vivo* (Hsia and Callis, 2010). Using a complementation assay, we showed that wild-type RING domains of each protein and the BRIZ1-BRIZ2 interaction domain are required for *in vivo* function (Hsia and Callis, 2010). We proposed that BRIZ1 and BRIZ2 function as subunits of a heteromeric E3 ligase.

Here, we further characterize the phenotype of loss-of-function *briz* mutants and provide evidence that *BRIZ1* and *BRIZ2* suppress ABA signaling in germination and early seedling growth. *briz* mutants do not have elevated endogenous ABA levels and their germination is hypersensitive to exogenous ABA. Reduction of ABA with a biosynthetic inhibitor allows some post-germination growth of *briz* embryos. *briz2* mutants exhibit improved germination and growth in *abi5-7* or *gin1-3* mutant backgrounds that have deficiencies in ABA signaling or synthesis, respectively. Similarly, *briz1* mutants exhibit increased germination in the *abi5-7* background. We also show that some *briz2-3 abi5-7* double mutants are able to grow to maturity and set seed. Seeds from *briz2-3 abi5-7* plants are hypersensitive to sucrose and glucose. These data support a model where the BRIZ E3 complex functions to suppress ABA responses during seed germination and post-germination seedling growth.

## Materials and Methods

### Plant Material and Growth Conditions

*Arabidopsis thaliana* ecotype Col-0 (CS70000), *briz1-1* (At2g42160, SALK_085207), *briz2-1* (At2g26000, SALK_094761), *briz2-2* (At2g26000, SALK_151060), *briz2-3* (At2g26000, FLAG_122B09), and *gin1-3* (At1g52340, CS6147) were obtained from the Arabidopsis Biological Resource Center in Columbus, Ohio^1^ and the *briz* lines were back-crossed at least four times to Col-0. The *abi5-7* allele (At2g36270, E74-1) as described in Nambara et al. (2002) was first obtained from Eiji Nambara and later re-acquired from Ruth Finkelstein (UC Santa Barbara).

Seeds were surface-sterilized in a solution of 25% commercial bleach and 0.1% Triton-X100 (Sigma, 93443) for 10 min, rinsed with sterile H_2_O, and stratified at 4°C for at least 24 hours (h) before plating. For bacto-agar-grown seedlings, seeds were plated on solid growth media (GM) consisting of 4.3 g/L Murashige and Skoog basal salt mixture (Sigma, M 5524), 1% sucrose (Fisher Scientific), 0.5 g/L MES (Calbiochem 475893), 1X B vitamins (0.5 μg/ml nicotinic acid, 1 μg/ml thiamine, 0.5 μg/ml pyridoxine, and 0.1 μg/ml myo-inositol, all from Sigma), and 8 g/L BD Bacto Agar (Fisher Scientific), pH 5.7. After 2 weeks at room temperature under constant light, seedlings were transplanted from agar GM plates to soil and plants were grown at 20°C with 50% humidity and 16 h light/8 h dark.

### Growth Media Modifications

A 100 mM fluridone (Chem Service, Inc. N-13217) stock in DMSO was diluted in GM to a final concentration of 100 μM. A 1 M brassinolide (BL; Santa Cruz Biotechnology, sc-391736) stock in DMSO was diluted in GM to a final concentration of 10 μM. Plates with 0.1% DMSO were used as solvent controls for fluridone and BL plates. A 10 mM ABA (Sigma, A-1049) stock in ethanol was diluted in GM to a final concentration of 0.1 μM. A 100 mM gibberellic acid (GA_3;_ Sigma, G-7645) stock in ethanol was diluted in GM to final concentrations of 10, 100, 200, or 1 mM. A 1 M aminocyclopropane carboxylic acid (ACC; Sigma, A-3903) stock in ethanol was diluted in GM to a final concentration of 50 μM. A 50 mM 2,4D (Sigma, D-8407) stock in ethanol was diluted in GM to a final concentration of 1 μM. Plates with 0.1% EtOH were used as solvent controls for ABA, GA_3_, 2,4-D, and ACC plates. GM plates with mannitol or glucose contained 1% mannitol or 1% glucose instead of 1% sucrose.

### Genotyping

Primers sequences for PCR genotyping are listed in Supplementary Table S1. For *briz1-1* lines, primer 9-097 was used with primer 9-098 to produce a WT gene-specific product, and primer 9-098 was used with the T-DNA left border primer 9-001 to produce a T-DNA junction product. For *briz2-1* lines, primer 6-674 was used with primer 6-675 to produce a WT gene-specific product, and primer 6-675 used with the T-DNA left border primer 9-001 to produce a T-DNA junction product. For *briz2-2* and *briz2-3* lines, primer 6-981 was used with primer 6-982 to produce a WT gene-specific product. For *briz2-2* lines, primer 6-982 was used with the T-DNA left border primer 9-001 to produce a T-DNA junction product. For *briz2-3* lines, primer 6-982 was used with the T-DNA left border primer 8-133 to produce a T-DNA junction product. For *abi5-7* lines, a dCAPs reaction was used. After amplification with primers abi5-7dCAPS-F and abi5-7dCAPS-R, products were digested with *Hin*fI and separated by 3% agarose gel electrophoresis. A second *Hin*fI site is present only in the *abi5-7* allele. For *gin1-3* lines, products generated with primers 6-745 and 6-746 were separated by 3% agarose gel electrophoresis. The *gin1-3* allele has a 50 bp deletion and these primers span the deletion site, making a shorter PCR product. PCR genotyping was used to identify F2-F4 individuals of the specified genotype.

### SCR/Germination Experiment

Age-matched seeds were used and were ~7 months post-harvest and the results were similar for seeds ~2 weeks post-harvest. Seed status was assessed at the designated times using forceps and a dissecting microscope in a sterile hood to be able to rotate each seed to see seed coat rupture (SCR) and radicle emergence (germination). The data represent the mean of three independent experiments, with three replicas of ~50 seeds per genotype per experiment.

### Viability Staining

Seeds from *BRIZ1/briz1-1*, *BRIZ2/briz2-1*, and *BRIZ2/briz2-2* plants were plated on GM plates and grown in a controlled environment (constant light, 22°C) for 15 or 30 days. Embryos that emerged from the seed coat were selected for the experiment. Sytox Orange nucleic acid stain in DMSO (Invitrogen, S11368) was diluted in water to a working concentration of 250 nM. Fluorescein diacetate (FDA) powder (Invitrogen, F1303) was dissolved in acetone to make a 5 mg/ml stock, then diluted in water to a working concentration of 5 μg/ml. Embryos were either left untreated or were heat-treated at 98°C for 5 min. Embryos were stained for 10–15 min before imaging with an Olympus Confocal Laser Scanning Microscope (model FV5-LDPSU). Sytox stained seedlings were imaged using the 10x lens and filter with laser excitation at 543 nm. FDA stained seedlings were imaged using the 10x lens and filter with laser excitation at 488 nm.

### ABA Determination

The two silenced lines were the T2 generation from two independent *briz2-1* lines initially complemented by expressing Myc-BRIZ2 protein (Hsia and Callis, 2010). Initially with 100% germination in a homozygous *briz2-1* background, progeny from some individuals showed 0% germination. Seeds were soaked in water for 1 h at room temperature and water was removed before flash freezing. ABA levels were then determined by the Danforth Center, St. Louis, MO^2^ as described in Chen et al. (2009) with D6ABA used as an internal standard. A mix of D6ABA and ABA were used for a standard curve.

### Embryo Excisions

Embryo excisions were performed as described in Lee et al. (2010b) and shown in Lee and Lopez-Molina (2013). Excised embryos were placed on sterilized 60 μM nylon net filters (Millipore #NY6002500) on top of GM solid media and photographed using a dissecting microscope. To identify *briz1-1* and *briz2-3* seeds, which are indistinguishable from wild-type at the dry seed stage, seeds from heterozygous *BRIZ1/briz1-1* or *BRIZ2/briz2-3* plants were plated on the appropriate growth media, and seeds that had not germinated after 48 h were selected for excision. Age-matched seeds were used.

### SDS-PAGE and Western Blotting

For visualization of Myc-BRIZ2 protein, seeds were plated on GM plates and stratified at 4°C for 24 h, then ground in buffer containing 50 mM Tris-HCl pH 7.5, 150 mM NaCl, 0.5% NP-40, 1 mM PMSF, and one EDTA-free protease inhibitor tablet (Roche, 1836153)/10 ml. Bio-Rad Protein Assay was used to quantify total protein. About 50 μg total protein was loaded on a 9% polyacrylamide gel for SDS-PAGE separation, and then transferred to a PVDF-P membrane (Immobilon). Membranes were blocked in 5% nonfat powdered milk (Carnation or Sunny Select from local grocery stores) dissolved in TBS + 0.1% Tween 20 (Sigma, P1379). Myc-tagged proteins were detected with Anti-Myc-mouse (Roche # 11667149001) followed by the secondary antibody peroxidase-conjugated AffiniPure Goat Anti-Mouse IgG (H + L; Jackson ImmunoResearch) at a dilution of 1:10,000. SuperSignal West Pico Chemiluminescent Substrate (Thermo Scientific) was used as a chemiluminescent substrate. Chemiluminescence was captured on X-ray film (Phenix).

For visualizing endogenous ABI5 protein, *abi5-7* seeds (dry), Col seeds (dry, stratified at 4°C for 24 h, or stratified at 4°C for 24 h and then plated on GM and grown under constant light at 22°C for the indicated times), and *briz1-1* and *briz2-3* seeds (stratified at 4°C for 24 h and then plated on GM and grown under constant light at 22°C for 72 h and identified by their non-germination) were ground in buffer containing 50 mM Tris-HCl pH 7.5, 250 mM NaCl, 0.5% NP-40, 1 mM PMSF, 50 μM MG132 (Peptides International), and one EDTA-free protease inhibitor tablet (Roche, 1836153)/10 ml. Lysates were collected after centrifugation at 13,000 rpm for 10 min at 4°C, and total protein concentrations were measured with Bio-Rad Protein Assay. About 25 μg total protein per sample was loaded on a 10% polyacrylamide gel for SDS-PAGE, and then transferred to a PVDF-P membrane (Immobilon). Membranes were blocked in 5% nonfat powdered milk dissolved in TBS + 0.1% Tween 20, as described above. ABI5 protein was detected with anti-ABI5 (gift from R. Vierstra, WU-STL) at a dilution of 1:5,000 followed by the secondary antibody peroxidase-conjugated AffiniPure Goat Anti-Rabbit IgG (H + L; Jackson ImmunoResearch) at a dilution of 1:10,000. ProSignal Dura (Prometheus # 20-301) was used as a chemiluminescent substrate. Chemiluminescence was digitally imaged in the linear range of detection using the ImageQuant LAS400 imaging system (GE).

### Seed Size Measurements and Protein Comparison

For seed size measurements (Supplementary Figure S7), seeds were placed on double-sided tape on a microscope slide and photographed through a dissecting microscope. *BRIZ2/briz2-3 abi5-7* and *BRIZ2/briz2-3 ABI5* were siblings from the cross used to generate the *briz2-3 abi5-7* double mutant plants. Seed length was measured using Image J. For seed weight measurements, for each genotype, three groups of 100 seeds were weighed. For embryo size measurements, embryos were excised as described above and measured using Image J. Measurements were analyzed using GraphPad Prism.

### RNA Extraction and Quantitative PCR

Seedlings were grown in liquid GM under constant light for 6 days and treated with 50 μM ABA or 0.1% ethanol as mock treatment for 6 h. Total RNA was isolated using the RNeasy Plant Mini Kit (Qiagen, 74903) according to manufacturer’s instructions. About 2.4 μg of total RNA was used in a 20 μl reverse transcription reaction performed with Superscript III reverse transcriptase (Invitrogen, 18080-044). Real-time PCR amplification was performed with 50 μl of reaction solution containing 1 μl of first-strand cDNA, 10 pmoles of each primer, and 1x SYBR Green Master Mix (Applied Biosystems, A46109). Relative transcript levels were obtained using the comparative Ct method. The experiment was performed independently three times with three technical replicates each time.

## Results

### *briz* Seeds Are Defective in Seed Coat Rupture and Germination, but *briz* Embryos Are Alive

**Figure 1:**
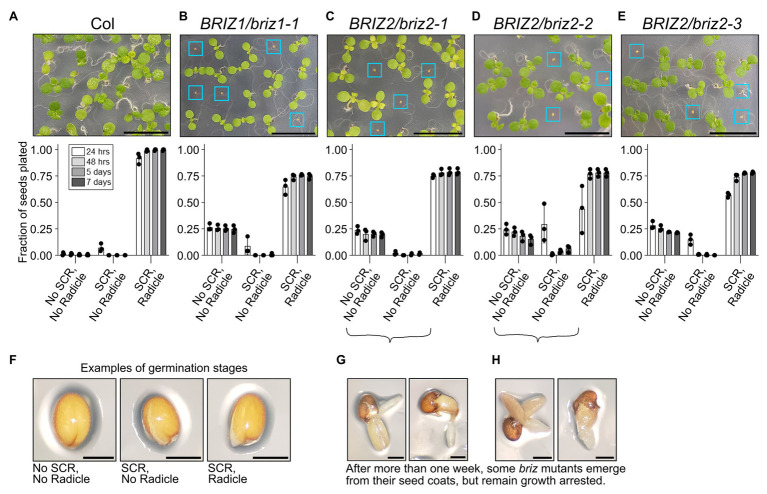
*briz1* and *briz2* mutants are defective in seed coat rupture (SCR) and germination and have arrested growth. (**A–E**, top) Top, image of a portion of a GM plate with seeds from plants of the following genotypes after ~7 days at 20°C under constant light: **(A)** wild-type Col, **(B)** *BRIZ1/briz1-1*, **(C)** *BRIZ2/briz2-1*, **(D)** *BRIZ2/briz2-2*, and **(E)** *BRIZ2/briz2-3*. Blue boxes surround *briz* seeds with non-germination or slow germination phenotypes. Scale bars represent 1 cm. (**A–E**, bottom) The fraction of total seeds at each of three different stages of germination was scored after 24 and 48 h, 5 and 7 days at 20°C constant light, and graphed for each genotype. Graphs show means of three independent experiments with approximately 150 seeds per genotype per experiment. Bars represent ±SD. Examples of each germination stage are shown in **(F)**. The three stages are (1) no SCR and no radicle emergence; (2) SCR but no radicle emergence; and (3) both SCR and radicle emergence (germination). After more than 1 week, some *briz* embryos emerge from their seed coats (germinate), but remain pale and growth arrested, examples in **(G)** and **(H)**. Scale bars in **(F–H)** represent 0.25 mm.

Previously we briefly described the effects of loss-of-function T-DNA insertions in *BRIZ1* and *BRIZ2* in *Arabidopsis* (Hsia and Callis, 2010). Here, we more closely analyze the behavior of *briz* homozygous seeds over time and assess their ability to initiate SCR separately from radicle emergence. SCR typically occurs before radicle emergence, with the latter process generally acknowledged as germination (reviewed in Holdsworth et al., 2008). Examples of the three phenotypic classes quantified in Figures 1A–E are shown in Figure 1F: no SCR, no radicle; SCR; and SCR, radicle. Under our growth conditions, age-matched wild-type (Col) seeds rapidly transverse through SCR and complete germination by 48 h (Figure 1A, “SCR, Radicle” class). Seeds from plants heterozygous for a T-DNA insertion in the coding region of either *BRIZ1* (*briz1-1*) or *BRIZ2* (*briz2-1*, *-2*, and *-3*) segregate for two strikingly different phenotypes in a 3:1 ratio. Three-fourths of the seeds exhibit a wild-type phenotype, largely completing SCR and germination by 48 h at 20°C. However, one-quarter of the seeds fail to exhibit SCR even after 7 days (Figures 1B–E, “No SCR, No radicle” class). Between 7 and 14 days, a few seeds exhibit SCR (although it is often abnormal, with ruptures randomly across the surface rather than directly along the root axis), and some of these continue on to germinate. However, these germinated seedlings do not accumulate visible levels of chlorophyll, their cotyledons fail to expand and reflex, and root elongation is minimal (Figures 1G,H). The *briz* mutant phenotype referred to in this work includes both non-germinated seeds, seeds with SCR only, and the pale, undeveloped, late germinating embryos shown in Figures 1G,H. All *briz1-1*, *briz2-1*, *briz2-2*, or *briz2-3* single mutants exhibit strongly delayed SCR and when germination occurs, have phenotypically similar seedlings that fail to progress in development.

**Figure 2:**
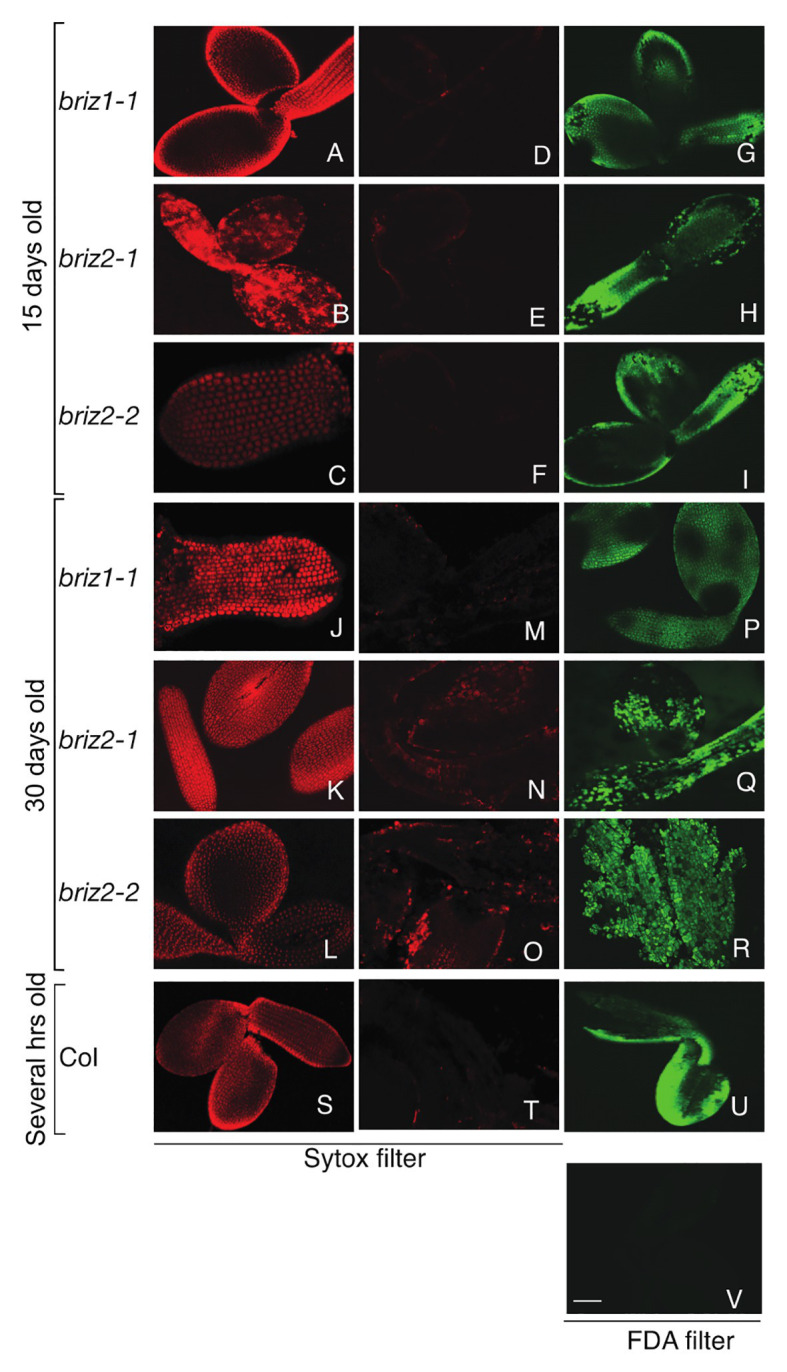
*briz1* and *briz2* mutants are alive despite arrested growth. Seeds from *BRIZ/briz* plants were plated on GM for 15 days **(A–I)** or 30 days **(J–R)** before embryos were treated with viability stains. Embryos from Col seeds soaked for 1 h in water were used as controls **(S–V)**. *briz* embryos were either heat-treated for 5 min at 98°C or untreated, followed by Sytox staining (**A–C**, **J–L**, **S** or **D–F**, **M–O**, **T**, respectively) or fluorescein diacetate (FDA) only staining **(G–I**, **P–R**, **U**,**V)** before confocal microscopy. Heat-treated **(S)** or untreated Col after Sytox **(T)** or FDA staining **(U)**. Heat-treated Col after FDA staining **(V)**. White bar **(V)** represents 70 μm. All were visualized at the same scale.

We hypothesized that the *briz* phenotype may result from embryo cell death and that passive absorbance of water could be sufficient for delayed SCR and late embryo protrusion from the seed coat. We used Sytox orange and FDA staining (Truernit and Haseloff, 2008) to test the viability of *briz* seedlings. Sytox orange penetrates non-viable cells and stains nuclei. FDA enters living cells and is converted to a fluorescent compound, fluorescein, by cytosolic esterases. Before staining and confocal microscopy, embryos were either left untreated or were killed with a brief 98°C heat treatment. As expected, control Col embryos did not stain with Sytox orange unless they were heat-treated (Figures 2T vs. S). Untreated Col embryos stained with FDA (Figure 2U), while heat-treated Col embryos did not (Figure 2V). These results show that cells in Col embryos are alive unless they are killed with heat treatment. Col seeds used in the experiment were soaked for a few hours in water because these seeds are morphologically similar to 15-day-old *briz* seeds.

Seeds from *BRIZ1*/*briz1-1*, *BRIZ2/briz2-1*, or *BRIZ2/briz2-2* plants were plated on GM for either 15 or 30 days before *briz* embryos were collected. Like Col embryos, 15-day-old *briz1* and *briz2* embryos did not stain with Sytox orange (Figures 2D–F) unless they were heat-treated prior to staining (Figures 2A–C). Untreated *briz1* and *briz2* embryos stained with FDA (Figures 2G–I). These results show that cells in 15-day-old *briz1* and *briz2* embryos are alive unless they are killed with heat treatment. The same tests were performed on 30-day-old *briz* embryos. Similar to 15-day-old embryos, 30-day-old *briz1* and *briz2* embryos were primarily Sytox orange negative (Figures 2M–O) unless heat-treated first (Figures 2J–L), and untreated embryos were FDA positive (Figures 2P–R). These results indicate that cells are alive in 30-day-old *briz* embryos even though the embryos are arrested at the germination/post-germination stage.

### The *briz* Phenotype Can Be Partially Rescued by the Carotenoid Biosynthetic Inhibitor Fluridone, and the Mutant Phenotype Returns With Addition of Low Levels of ABA

**Figure 3:**
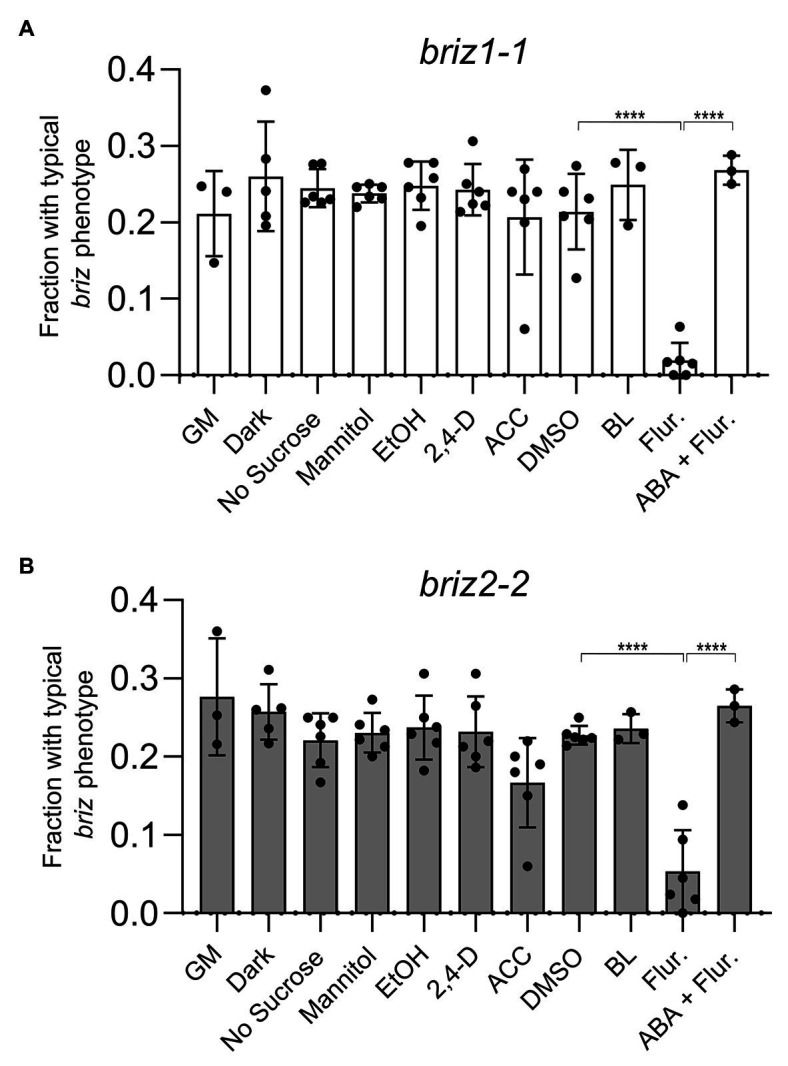
Fluridone reduces the fraction of *briz1-1* and *briz2-2* mutants with a typical *briz* phenotype, which returns upon addition of abscisic acid (ABA). **(A)** Seeds from heterozygous *BRIZ1/briz1-1* plants were plated on agar GM plates or on GM plates lacking sucrose or containing 1% mannitol, 1 μM 2,4-D, 50 μM aminocyclopropane carboxylic acid (ACC), 10 μM brassinolide (BL), 100 μM fluridone (Flur.), or 100 μM fluridone + 0.1 μM ABA. Plates with 0.1% EtOH were used as solvent controls for the 2,4-D and ACC plates. Plates with 0.1% DMSO were used as solvent controls for the BL and fluridone plates. For the dark treatment, GM plates were wrapped in foil. Seedlings with a typical *briz* phenotype were scored after 12–15 days and represented as a fraction of the total seeds plated. Asterisks (****) represent *p* < 0.0001, ANOVA analysis (Graph Pad Prism). Bars represent ±SD. Data represent 3–4 independent experiments with 30–50 seeds each. **(B)** Seeds from *BRIZ2/briz2-2* plants, analyzed as described in **(A)**.

Environmental conditions were sought that would promote germination and/or growth of *briz* embryos. Because single homozygous *briz* individuals cannot be grown to maturity for seed production, all studies were conducted on seeds from selfed heterozygous *BRIZ/briz* parents. When seeds from *BRIZ1/briz1-1* or *BRIZ2/briz2-2* plants were plated on GM alone or on GM containing the solvent controls DMSO or ethanol, ~0.25 of the seeds exhibited the *briz* phenotype (Figure 3; Supplementary Figure S1), as expected for the segregation of a single recessive trait (also see Figure 1). Chi-square goodness of fit tests confirmed that phenotypic segregation of seeds from heterozygous *BRIZ1/briz1-1* and *BRIZ2/briz2-2* plants fit an expectation of 3:1 wild-type:*briz*, with *p*-values of 0.18 and 0.35, respectively (*n* = ~220 seed).

We tested the effects of light or various additions/modifications to GM plates and predicted that conditions that rescued the *briz* phenotype would reduce the fraction of seeds with a typical *briz* phenotype. This fraction was unaffected by incubating the plates at RT in the dark (Figures 3A,B). Neither the absence of sucrose nor the addition of mannitol had a significant effect on the fraction of *briz* seedlings (Figures 3A,B). Similarly, the fraction of *briz* seedlings was unaffected by addition of the synthetic auxin 2,4,dichlorophenoxyacetic acid (2,4 D), the ethylene precursor ACC, or BL (Figures 3A,B; see Supplementary Figure S1 for example of BL plates). The results were the same for seeds segregating for either *briz1-1* (Figure 3A) or *briz2-2* (Figure 3B).

By contrast, addition of fluridone, a carotenoid biosynthetic inhibitor that also blocks ABA biosynthesis (Bartels and Watson, 1978), resulted in a significant reduction of phenotypically *briz* embryos (Figures 3A,B). Similar results were observed for a second *briz2* allele, with seeds from a *BRIZ2*/*briz2-1* parent (Supplementary Figure S2; for examples of plates for all mutants, see Supplementary Figure S3A, which are non-green because of insufficient chlorophyll-protecting carotenoids, resulting in photo-oxidative damage to chlorophylls, Bartels and Watson, 1978). These results demonstrate that fluridone promotes further development of homozygous *briz* seedlings. However, few *briz* seedlings had expanded visible true leaves, and those that did were smaller than their wild-type siblings, indicating that suppression of the *briz* phenotype was incomplete.

To determine whether fluridone’s ability to suppress the *briz* phenotype is caused by a reduction in endogenous ABA and not a reduction in some other carotenoid-related metabolite, we tested the effect of including 0.1 μM ABA in the GM plates in addition to fluridone. While this concentration of ABA did not inhibit germination of wild-type Col seeds (Supplementary Figure S3B), it strongly affected germination of a subset of seeds from *BRIZ/briz* parents. Around 25% of the seeds failed to germinate and looked identical to the *briz* seeds grown on GM plates (Figure 3). These results suggest that the growth-promoting effect of fluridone on *briz* seeds results from lowering endogenous ABA levels, not from affecting the levels of other products derived from the carotenoid biosynthetic pathway.

### *briz* Seeds Do Not Hyper-Accumulate ABA

**Figure 4:**
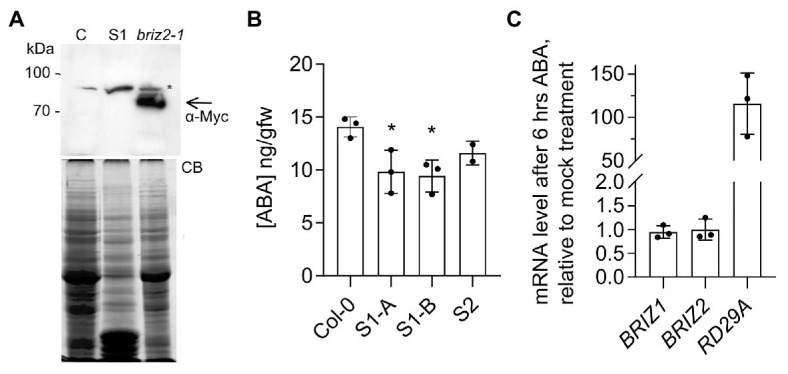
*briz* seeds do not hyper-accumulate ABA, and ABA does not affect *BRIZ* mRNA levels. **(A)** Anti-Myc immunoblot of extracts from 24 h imbibed seeds from untransformed control Col (left), a complemented *briz2-1* line expressing Myc-BRIZ2 with a wild-type phenotype (right), and the homozygous S1 *briz2-1* line with the silenced *Myc-BRIZ2* transgene, whose progeny failed to germinate (S1, middle lane). Below, total protein staining with Coomassie blue (CB). Asterisk denotes a non-specific immunoreactive band. Arrow denotes position of Myc-tagged BRIZ2. **(B)** ABA content [ng/gm fresh weight GFW)] in 1 h imbibed seeds from wild-type (Col) and two silenced lines (S1 and S2) derived from independent T1 briz2-1 *Myc-BRIZ2* transgene-containing complementation lines (Hsia and Callis, 2010). S1-A and S1-B represent siblings of same initial transformation event. Mean of three replicates except for S2, which was measured twice, ±SD. ANOVA was performed for S1 compared to Col (GraphPad Prism, with Tukey’s multiple comparisons test). **(C)** Expression of *BRIZ1*, *BRIZ2*, and *RD29A* mRNAs after 6 h of ABA or mock treatment as assayed by quantitative RT-PCR normalized to *UBQ10*. Results are shown as mean ± SD (*n* = 9) from three independent experiments. *RD29A* serves as a positive control for ABA response.

*briz* seeds phenotypically resemble wild-type seeds plated on a high concentration of exogenous ABA (Leung et al., 1994). One possible explanation for the strong growth-arrested phenotype (Figures 1, 2) and hypersensitivity to added ABA (Figure 3) seen in *briz* mutants could be an elevated level of endogenous ABA. To test whether *briz* seeds have higher (ABA) than wild-type seeds, we measured (ABA) in two lines whose progeny exhibited a 100% *briz* phenotype. These lines arose from two independent transgenic lines that initially expressed an epitope tagged form of BRIZ2 (Myc-BRIZ2) in the *briz2-1* background (Hsia and Callis, 2010). In subsequent generations, 100% of the seeds exhibited the *briz* phenotype, suggesting that both the endogenous *BRIZ2* gene and the *Myc-BRIZ2* transgene had silenced. Western blots of seed protein extracts verified that non-germinating seeds from one line (S, for silenced) did not express Myc-BRIZ2 protein encoded by the transgene, while Myc-BRIZ2 protein was readily visible in an independent line that carried the same transgene and exhibited 100% germination (Figure 4A). Seeds from the two silenced lines and age-matched Col were hydrated for 1 h, after which ABA content was determined. *briz* seeds did not have a significantly higher level of ABA than the wild-type control, and in fact S1 had a slightly lower ABA level (Figure 4B). Analysis of ABA content in *briz1-1* seeds was not possible because we could not obtain silenced lines in the *briz1-1* mutant background that gave the 100% *briz* seeds needed for the analysis. Altogether these data suggest that briz embryos do not hyper-accumulate ABA, and that *briz* embryos are hypersensitive to both endogenous and exogenous ABA.

### ABA Does Not Affect *BRIZ* mRNA in Seedlings

To evaluate whether ABA affects *BRIZ* expression, we used qPCR to measure relative *BRIZ* mRNA levels in 6-day-old Col seedlings treated with for 6 h with ABA. Exogenous ABA induces robust transcriptional responses at this developmental stage (Lopez-Molina et al., 2001). Expression of *RD29A*, a well-known ABA-responsive gene (Nakashima et al., 2006), increased by approximately ~100-fold after ABA treatment, indicating a strong response to ABA (Figure 4C). However, neither *BRIZ1* nor *BRIZ2* mRNA levels were affected by ABA treatment (Figure 4C).

### *briz2-2* gin1-3 Double Mutants Have Increased Germination, Greening, and Growth

**Figure 5:**
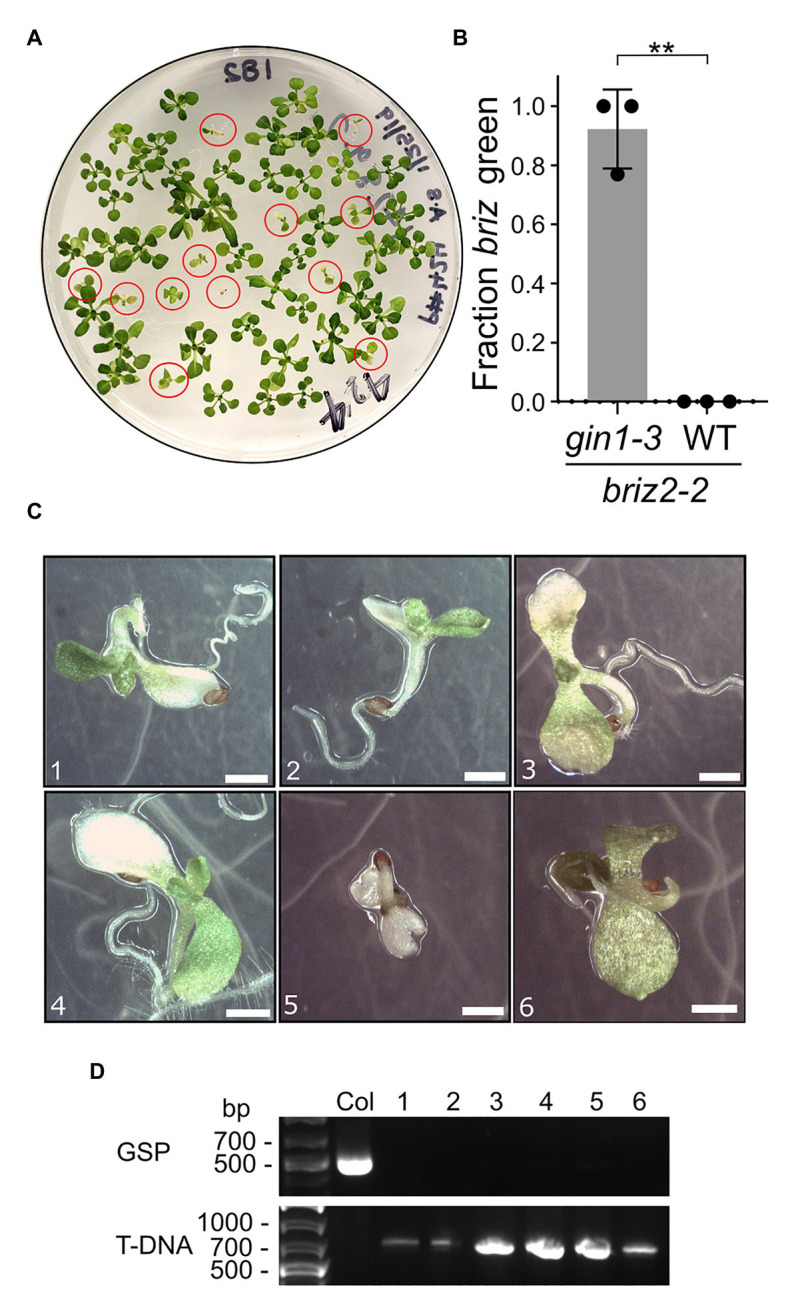
*briz2-2* mutants have increased germination, greening, and growth in the *gin1-3* background. Seeds from plants homozygous for *gin1-3* and heterozygous for *BRIZ2/briz2-2* were plated on plain GM. **(A)** Plate with seeds from a *gin1-3* plant heterozygous for *BRIZ2/briz2-2*. Red circles are *briz2-2 gin1-3* seedlings. **(B)** Fraction of homozygous *briz2-2* mutants with green expanded cotyledons was recorded after 14 days. *N* = 3 plates with approximately 50 seeds per plate. Bars represent ±SD. Asterisks indicate a significant difference (** for *p* < 0.01) according to a *t*-test with Welch’s correction in GraphPad Prism. **(C)** Photos of homozygous *briz2-2* seedlings at 18 days. Bars, 1 mm. **(D)** PCR genotyping of seedlings in panel **(C)** for *BRIZ2*, with Col as a WT control. GSP = PCR with gene-specific primers flanking the T-DNA insertion site. T-DNA = PCR with T-DNA primer and gene-specific primer. Primer sequences in Supplementary Table S1.

To test whether the *briz* phenotype can be suppressed if endogenous ABA is reduced genetically, *BRIZ2/briz2-2* plants were crossed with ABA-deficient *gin1-3* plants. *GIN1* (also called *ABA2*) encodes a short-chain dehydrogenase/reductase enzyme involved in ABA biosynthesis (Cheng et al., 2002). *gin1-3* is a null allele with a deletion in the second exon which results in an early stop codon, and *gin1-3* plants have low levels of endogenous ABA (Cheng et al., 2002). Seeds from plants homozygous for *gin1-3* and heterozygous for *BRIZ2/briz2-2* were plated on GM and they segregated for a reduced fraction of seeds/seedlings with a *briz* phenotype (*χ*^2^ = 29, *p* < 0.0001 for 3:1 WT:*briz*) compared to seeds from *BRIZ2*/*briz2-2* heterozygous plants (*χ*^2^ = 0.007, *p* = 0.93). Seeds from a *gin1-3* single mutant sibling had nearly 100% germination and greening (Supplementary Figure S4A). Exogenous ABA restored the 3:1 ratio of WT:*briz* phenotype in the seeds from *BRIZ2/briz2-2 gin1-3* plants (*χ*^2^ = 0.06, *p* = 0.8; Supplementary Figure S4A), indicating that low ABA resulting from the *gin1-3* mutation allowed the additional growth of *briz* mutants. *briz2-2 gin1-3* seeds were identified by their slower germination and growth (circled in red, Figure 5A). After 2 weeks, an average of 92.3% of the *briz*2-2 *gin1-3* mutants had greened (Figure 5B) and most had developed true leaves (Figures 5C,D). However, *briz2-2 gin1-3* plants remained small and most eventually died on the GM plates or wilted quickly and died after they were transplanted to soil. The loss of *GIN1* likely contributed to the poor survival of the double mutants because *gin1-3* single mutants are smaller than wild-type plants and wilt quickly (Cheng et al., 2002) and were challenging to grow to maturity under our growth conditions. A few plants that were transplanted to soil were verified by PCR to be homozygous for *briz2-2* (Supplementary Figure S4). These results show that a reduction in endogenous ABA allows further development of *briz2-2* seedlings and support the hypothesis that *briz2-2* seedlings are growth arrested due to ABA hypersensitivity.

### In the *gin1-3* Background, Heterozygous *BRIZ1/briz1-1* Plants Produce More Underdeveloped Seeds Than Their WT *BRIZ* Sibs

*BRIZ1/briz1-1* plants were also crossed with *gin1-3* plants, and seeds from plants homozygous for *gin1-3* and heterozygous for *BRIZ1/briz1-1* were plated on GM. All 80 seeds germinated and looked wild-type. This was surprising, as we expected approximately 25% (the *briz1-1* homozygotes) to have a slow growth phenotype similar to *briz2-2 gin1-3* double mutants (see Figure 5). All seedlings were genotyped and only three possible *briz1-1* homozygotes were identified (Supplementary Figure S5A). These numbers do not fit the expected ratio of 1:2:1 WT:het:mutant for *BRIZ1* (*X*^2^ = 28.189, *p* < 0.0001). Instead, they fit a ratio of 1:2 WT:het (*X*^2^ = 0.311, *p* = 0.577), with the *briz1-1 gin 1-3* double mutant seeds frequency much reduced. Both male and female homozygous *briz1-1* gametophytes are still viable in the *gin1-3* background (if either gametophyte is not viable, a 1:1 WT:het ratio would be expected, and these data do not fit that ratio, with *X*^2^ = 8.165 and *p* = 0.004). In a larger plating of 300 seeds, only 12 seeds did not germinate (presumably homozygotes for *briz1-1*), well below the expected number of 75. These results suggest that many homozygous *briz1-1* seeds in the *gin1-3* background do not develop to the mature seed stage.

To investigate why there were fewer than expected *briz1-1* homozygotes, we examined siliques of heterozygous *BRIZ1/briz1-1* plants in the *gin1-3* background. Siliques of *BRIZ1/briz1-1 gin1-3* plants contained more undeveloped or collapsed seeds (40.2%) than *gin1-3* plants that were wild-type for *BRIZ1* (24.6%; Supplementary Figure S5B). *gin1-3* single mutants have defects in seed development and have many undeveloped seeds (Cheng et al., 2002), so our observation of 24.6% undeveloped seeds in *gin1-3* plants that were wild-type for *BRIZ1* was not surprising. These results suggest that in addition to seed loss due to *gin1-3*, many homozygous *briz1-1* mutant seeds in the siliques of heterozygous *BRIZ1/briz1-1* plants do not develop.

Despite the lower than expected number of homozygous *briz1-1* seeds, we were able to identify one homozygous *briz1-1* seedling (Supplementary Figures S5C,D). The seed took more than 1 week to germinate, and development was very slow (the photo in Supplementary Figure S5C was taken 26 days after plating). The seedling eventually developed four true leaves but died after it was transplanted to soil.

### GA_3_ Increases Germination of *briz* Mutants

**Figure 6:**
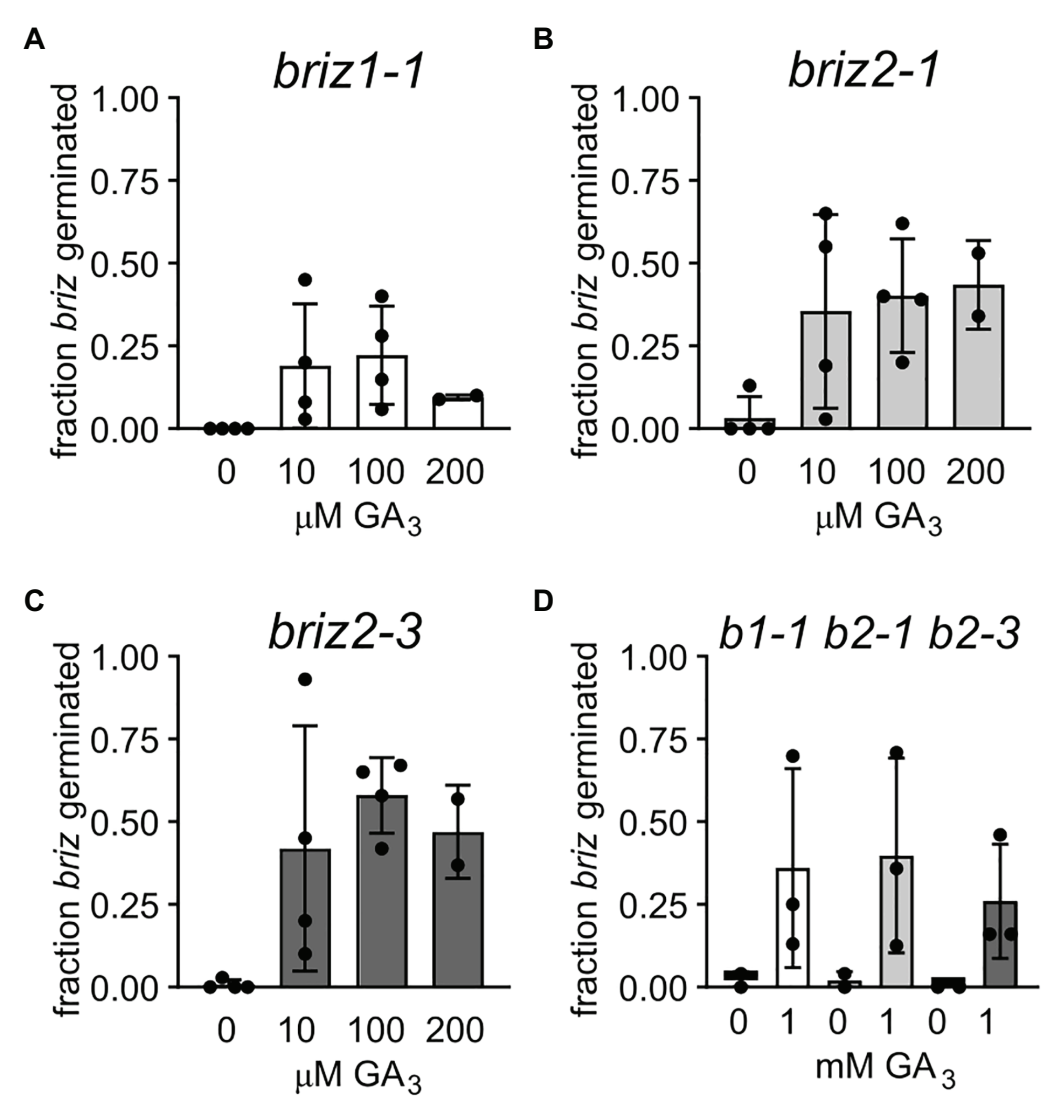
Gibberellic acid addition improves germination of *briz1-1*, *briz2-1*, and *briz2-3* mutants. **(A)** Seeds from plants heterozygous for *BRIZ1/briz1-1* were plated on GM with 0, 10, 100, or 200 μM GA_3_. Germination of *briz* seeds was scored after 1 week. Bars represent ±SD. Data from 2 to 4 independent experiments with 120–150 seeds per experiment. **(B)** Seeds from plants heterozygous for *BRIZ2/briz2-1*. **(C)** Seeds from plants heterozygous for *BRIZ2/briz2-3*. **(D)** Seeds were plated on GM with or without 1 mM GA_3_.

Abscisic acid and gibberellic acid (GA) act antagonistically during germination, so the ability of exogenous GA to affect the *briz* phenotype of delayed or no germination, or arrested seedling growth was tested. Seeds from heterozygous *BRIZ1/briz1-1*, *BRIZ2/briz2-1*, or *BRIZ2/briz2-3* plants were plated on GM or GM containing GA_3_. Homozygous *briz* seeds were identified after f5 days by their slower germination and the percent of the *briz* class of seeds that germinated was scored at 1 and 2 weeks (if all *briz* seeds germinated, the percent is 100%). After 1 week on GM, 0% of homozygous *briz1-1* seeds, 3.3% of homozygous *briz2-1* seeds, and 0.8% of homozygous *briz2-3* seeds had germinated. On GM plates containing 10 μM GA_3_, these percentages increased to 19.0, 35.5, and 42.0%, respectively (Figures 6A–C). Increasing the concentration of GA_3_ to 100 μM, 200 μM (Figures 6A–C), or 1 mM (Figure 6D) did not greatly increase germination above that observed for 10 μM GA_3_.

### *briz* Embryos Are Hypersensitive to Sucrose and Glucose and Elongation Can Be Promoted by GA_3_

**Figure 7:**
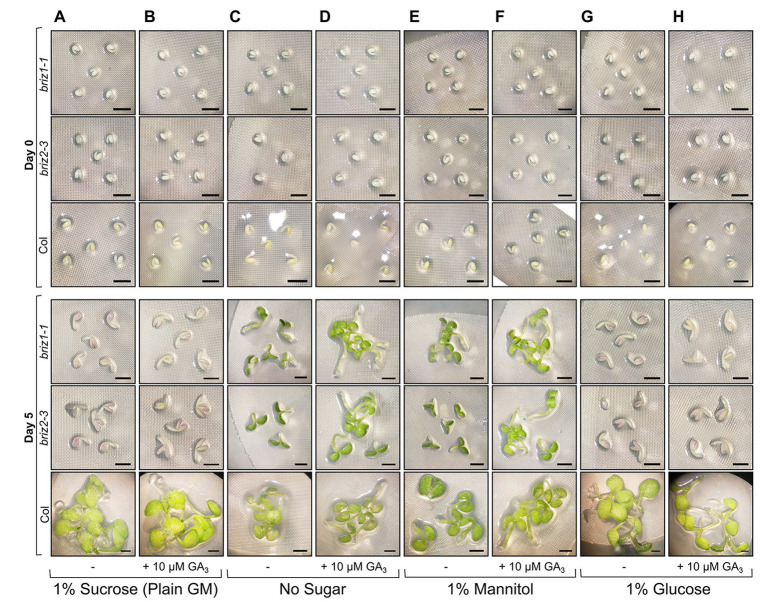
*briz* embryos are hypersensitive to sucrose and glucose. Embryos were excised from Col, *briz1-1*, or *briz2-3* seeds, placed on nylon filters on top of the indicated solid growth media (GM), and photographed at 0 days (top three rows) and 5 days (bottom three rows) after incubation at 20°C constant light. Four types of GM were used in the experiment, +/− 10 μM gibberellic acid (GA_3)_: **(A,B)** GM which contains 1% sucrose, **(C,D)** GM without sugar, **(E,F)** GM with 1% mannitol instead of sucrose, or **(G,H)** GM with 1% glucose instead of sucrose. Bars, 1 mm. At least three groups of embryos were plated for each genotype and type of growth media, and representative images are shown. A similar experiment with older seeds is shown in Supplementary Figure S7. Note: to identify *briz1-1* and *briz2-3* seeds, which are indistinguishable from wild-type at the dry seed stage, seeds from heterozygous *BRIZ1/briz1-1* or *BRIZ2/briz2-3* plants were plated on the appropriate growth media, and seeds that had not germinated after 48 h were selected for the experiment. PCR genotyping after the above experiment confirmed that these seeds were *briz1-1* or *briz2-3* homozygous mutants (shown in Supplementary Figure S6).

The effects of exogenous hormones on the embryo can be blocked if the seed coat remains intact. Because *briz* seeds are slow to exhibit SCR, we excised embryos from their seed coats to better assess the effects of exogenous GA_3_ on embryo growth. To identify homozygous *briz* seeds for the experiment, which cannot be distinguished at the dry seed stage, seeds from heterozygous *BRIZ/briz* plants were plated for 48 h on the types of GM plates described in Figure 7, and non-germinated seeds were selected. PCR genotyping after the experiment confirmed that these were *briz1-1* or *briz2-3* mutants (Supplementary Figure S6). Embryos removed from their seed coats and placed on GM (plain GM contains 1% sucrose) for 5 days remained growth arrested, though an increase in size and anthocyanin was visible (Figure 7A), and the addition of GA_3_ had no effect (Figure 7B). Glucose had the same effect as sucrose, inhibiting greening and growth (Figures 7G,H). When placed on media without sugar (Figure 7C) or with 1% mannitol as an osmotic control (Figure 7E), cotyledon greening and expansion was visible after 5 days in both *briz1-1* and *briz2-3* embryos. On both medias, *briz* hypocotyls elongated with added GA_3_, while the roots remained short (Figures 7D,F). Wild-type Col embryos greened on all media types and had elongated hypocotyls on medias containing GA_3_ compared to the same medias without GA_3_ (Figure 7, bottom row). These results indicate that compared to Col, *briz1-1* and *briz2-3* single mutants are hypersensitive to the sucrose concentration present typically in GM (1%). A combination of excision from the seed coat and a lack of sucrose in growth media allows *briz* mutants to develop more than previously observed, and to better respond to GA_3_. Seed age likely affects *briz* responsiveness to lack of sucrose, as seeds stored at RT for >6 months showed reduced greening on media without sucrose (Supplementary Figure S7).

### ABI5 Levels Are Higher in *briz* Seeds

**Figure 8:**
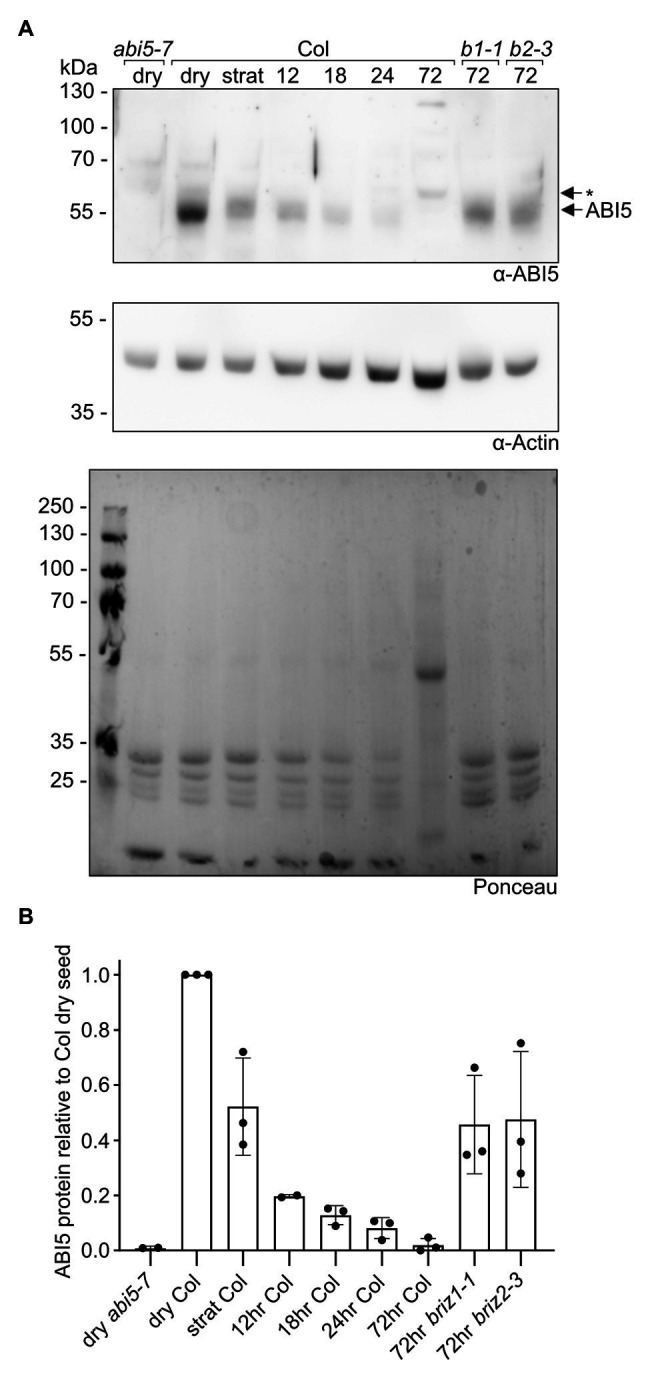
*briz* mutants have more ABI5 protein than wild-type seedlings at 72 h post-imbibition. Total proteins were extracted from Col seeds at various timepoints (dry, stratified at 4°C for 24 h, or stratified at 4°C for 24 h followed by incubation at RT under lights for 12, 18, 24, or 72 h), and from *briz1-1* and *briz2-3* seeds stratified at 4°C for 24 h followed by incubation at RT under lights for 72 h (seeds from heterozygous *BRIZ1/briz1-1* or *BRIZ2/briz2-3* plants were plated on GM, and seeds that had not germinated after 72 h were selected for the experiment). Around 25 μgs of total protein per sample were separated by SDDS-PAGE, and (**A**, top panel) anti-ABI5 western blotting was used to visualize the endogenous ABI5 protein in each sample. Dry *abi5-7* seeds (left lane) are a negative control for the anti-ABI5 antibody. (**A**, middle panel) As a loading control, an anti-actin western blot was performed on the same membrane. (**A**, lower panel) Ponceau staining of total proteins on the membrane. Note the decrease in seed storage proteins (< the 35 kDa marker) in the Col samples across timepoints, and the appearance of the Rubisco large subunit (near the 55 kDa marker) in the 72 h sample. Asterisk indicates non-specific band visible in 3-day-old seedings, but not dry seeds, from *abi5-7* null line. **(B)** ABI5 was normalized to actin, and the fraction of ABI5 protein relative to the amount in Col dry seeds was graphed for each sample. Graph shows the means of three independent experiments (see Supplementary Figure S8 for images of the other two experiments). Bars represent ±SD.

ABI5 is a bZIP transcription factor that is important for ABA responses during germination (Lopez-Molina et al., 2002). Given the strong growth arrest and ABA hypersensitivity without increased endogenous ABA in *briz* mutants, we asked whether ABI5 levels are higher in *briz* embryos. Seeds from *BRIZ1/briz1* or *BRIZ2/briz2-3* plants were plated on GM, stratified for 24 h at 4°C, and then incubated at RT for 72 h to identify *briz* seeds by their non-germination status. Protein extracts from these seeds were compared to protein extracts from wild-type seeds, either dry, stratified, or stratified and then incubated at RT for various timepoints (Figure 8; Supplementary Figure S8). In wild-type seeds, ABI5 levels fell after 24 h of stratification at 4°C, and continued to fall after the seeds were moved to RT. By 72 h, ABI5 levels were greatly reduced. By contrast, ABI5 protein was still visible in *briz1-1* and *briz2-3* seeds after 72 h at RT. These data indicate a correlation between lack of greening and growth and visible ABI5 protein levels in *briz* seeds.

### In the *abi5-7* Background, Both *briz1* and *briz2* Mutants Have Increased Germination, Greening, and Growth

**Figure 9:**
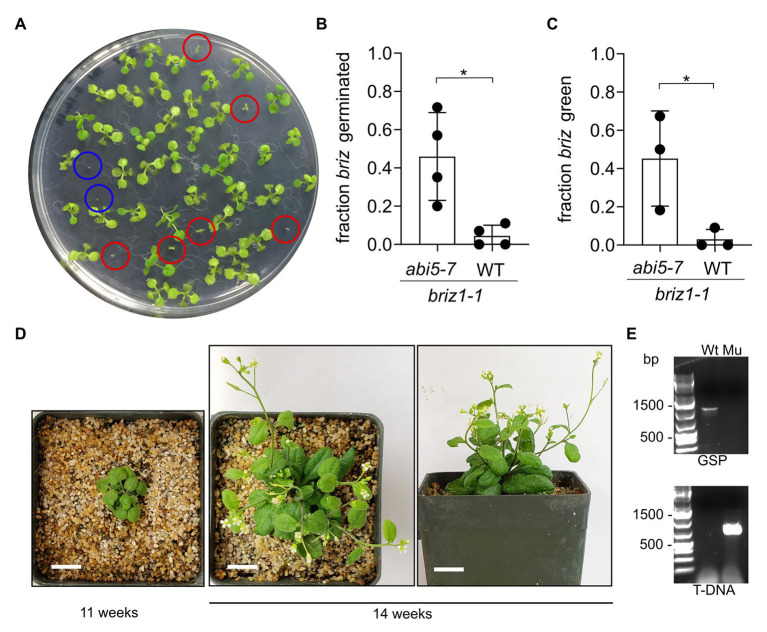
*briz1-1* mutants exhibit increased germination and greening in the *abi5-7* background. **(A)** A plate with seeds from an *abi5-7* plant heterozygous for *BRIZ1/briz1-1* incubated for 14 days constant light. Red circles are *briz1-1 abi5-7* seedlings with green cotyledons. Blue circles are typical non-germinated *briz1-1 abi5-7* seeds. **(B)** The fraction of germinated homozygous *briz1-1* seeds was recorded after 14 days. *N* = 4 experiments with 13 total plates with ~50 seeds per plate. Bars represent ±SD. **(C)** The fraction of homozygous *briz1-1* seedlings with green cotyledons was recorded after 14 days. *N* = 3 experiments with nine total plates with ~50 seeds per plate. Bars represent ±SD. **(D)** Photos of one *briz1-1 abi5-7* plant, 11 and 14 weeks after plating. Bars, 1 cm. **(E)** PCR genotyping of the plant in panel **(D)** for *BRIZ1*. Wt = wild-type control. Mu = the plant in **(D)**. Top panel, PCR with gene-specific primers flanking the T-DNA insertion site. Lower panel, PCR with a T-DNA primer and a flanking gene-specific primer. Asterisks indicate significant differences (* for *p* < 0.05) according to *t*-tests in GraphPad Prism. For **(B)** Welch’s correction was used.

**Figure 10:**
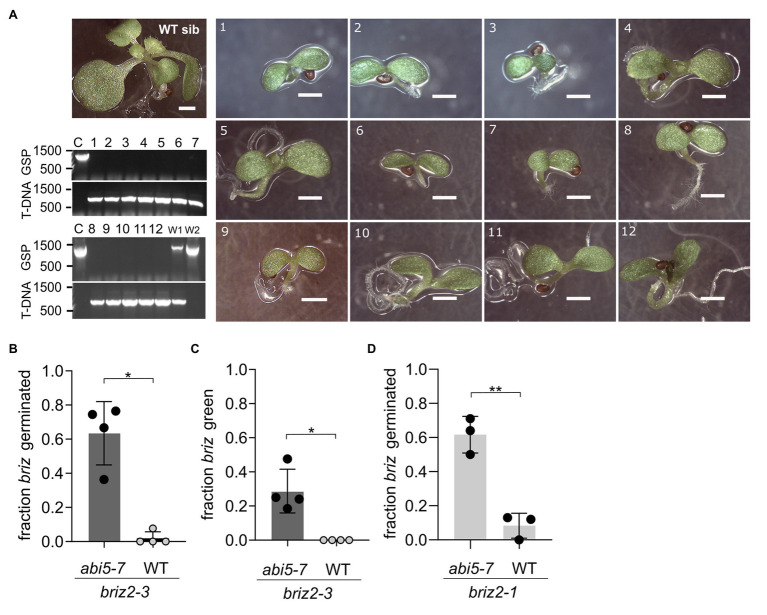
Multiple *briz2* mutants exhibit increased germination, greening, and growth in the *abi5-7* background. **(A)** Photos of homozygous *briz2-3 abi5-7* seedlings after 14 days compared to a phenotypically wild-type sibling on the same plate (top left, WT sib). Bars, 1 mm. Lower left, PCR genotyping of the pictured seedlings for *BRIZ2* (lane numbers correspond to numbers in photos). Top panel, PCR with gene-specific primers flanking the T-DNA insertion site. Lower panel, PCR with T-DNA primer and a flanking gene-specific primer. **(B)** Fraction of germinated homozygous *briz2-3* seeds after 14 days. *N* = 4 experiments with nine total plates, with ~70 seeds per plate. **(C)** Fraction of homozygous *briz2-3* seedlings with green cotyledons after 14 days. *N* = 4 experiments with nine total plates, with ~70 seeds per plate. **(D)** Fraction of germinated homozygous *briz2-1* seeds (a different allele than in panels **A–C**) after 14 days. *N* = 3 experiments with seven total plates, with ~50 seeds per plate. Bars represent ±SD **(B–D)**. Asterisks indicate significant differences (* for *p* < 0.05, ** for *p* < 0.005) according to *t*-tests in GraphPad Prism. For **(B–D)**, Welch’s correction was used.

To test whether ABI5 protein contributes to the growth arrest of *briz* embryos, we genetically eliminated *ABI5*. *BRIZ1/briz1-1*, *BRIZ2/briz2-1*, and *BRIZ2/briz2-3* plants were crossed with *abi5-7* plants. The *abi5-7* null allele contains a point mutation resulting in an early stop codon, and *abi5-7* seeds exhibit reduced sensitivity to ABA (Nambara et al., 2002). F3 seeds from F2 homozygous *abi5-7* plants also heterozygous for *BRIZ1/briz1-1*, *BRIZ2/briz2-1*, or *BRIZ2/briz2-3* were plated on GM. *briz1-1, briz2-1*, and *briz2-3* mutants in the *abi5-7* background had increased germination compared to the same alleles in wild-type *ABI5* F3 siblings, though they grew more slowly than wild-type seedlings (Figures 9A, 10A). PCR genotyping confirmed that these small, slowly developing, green seedlings were double mutants (Figures 9, 10). We were able to recover two *briz1-1 abi5-7* plants. Both grew to maturity on soil but produced few seeds, which failed to germinate (Figures 9D,E; Supplementary Figure S9).

F3 plants homozygous for *abi5-7* and heterozygous for *BRIZ2/briz2-3* were propagated to produce F4 seeds. Double mutant F4 seedlings with true leaves were transferred from plates to either soil or magenta boxes with GM. A subset survived to flower and produce seeds (Supplementary Figure S10). These plants produced leaves slowly and had small rosettes.

### Seeds From *briz2-3* *abi5-7* Plants Are Hypersensitive to Sucrose and Glucose When Compared to Seeds From *BRIZ2/briz2-3 abi5-7* Plants

Homozygous *briz2-3 abi5-7* plants (Supplementary Figure S10) produced seeds. Interestingly, these seeds were longer and weighed more and their embryos were longer than seeds from wild type (Col) and single mutant *abi5-7* plants (Supplementary Figure S11).

**Figure 11:**
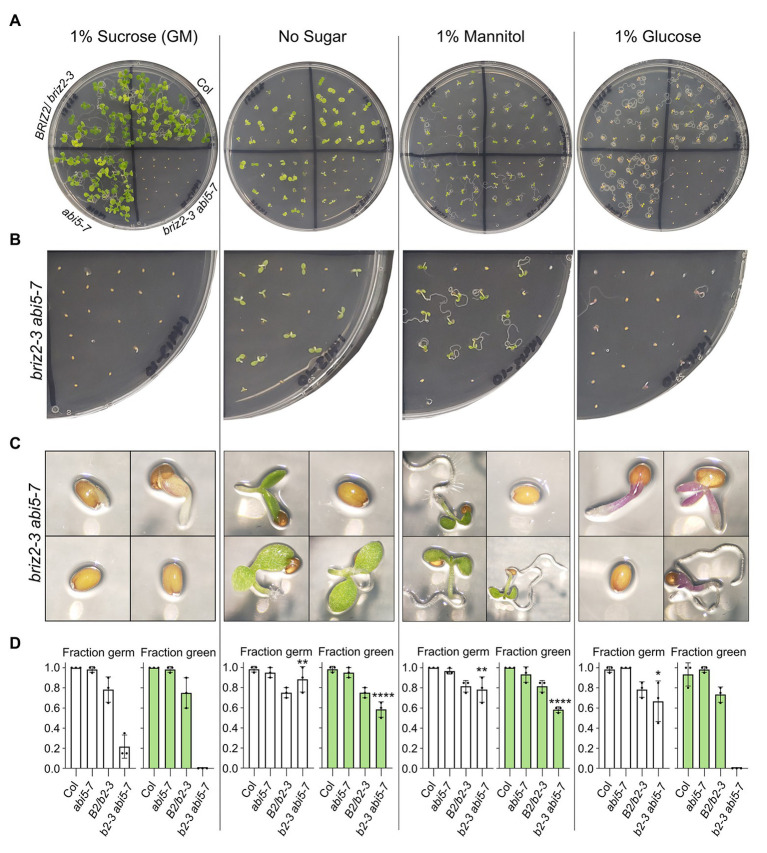
*briz2-3 abi5-7* mutants are hypersensitive to sucrose and glucose. Seeds from Col, *BRIZ2-3/briz2-3*, *abi5-7*, or *briz2-3 abi5-7* plants were plated on GM containing either 1% sucrose (left), no sucrose (2nd from left), 1% mannitol (2nd from right), or 1% glucose (right). All data shown are after 10 days of growth in constant light **(A)** Photos of plates. **(B)** Closer view of the *briz2-3 abi5-7* section on same plates. **(C)** Photos of individual seeds or seedlings from the *briz2-3 abi5-7* section from same plates. **(D)** Germinated (radicle emergence) seeds (white graphs) and germinated seeds with green expanded cotyledons (green graphs) were scored 10 days after plating. *N* = 3 independent experiments, with 20 seeds of each genotype per experiment. Bars represent ±SD. Asterisks indicate significant differences (* for *p* < 0.05, ** for *p* < 0.005, and **** for *p* < 0.0001, according to ANOVAs in GraphPad Prism) compared to the same genotype on GM (1% sucrose).

When seeds from the *briz2-3 abi5-7* plants were plated on GM, germination was slow, as previously observed with *briz2-3 abi5-7* seeds derived from heterozygous *BRIZ2/briz2-3 abi5-7* plants (see Figure 10), but surprisingly, after 10 days, the germinated seedlings did not have green cotyledons on GM (with 1% sucrose; Figure 11A). To directly test whether the sucrose present in GM inhibits germination and growth of seeds from double mutant *briz2-3 abi5-7* plants as observed previously with *briz* single mutant embryos, seeds were also plated on GM without sugar, GM with 1% mannitol instead as an osmotic control, or with 1% glucose instead of sucrose (Figure 11). Seeds from Col, *abi5-7*, and *BRIZ2/briz2-3* plants were plated as controls. After 10 days, only 21.7% of *briz2-3 abi5-7* seeds had germinated on GM containing sucrose, significantly lower than 88.3, 78.3, and 66.7% on GM lacking sugar, containing mannitol, or containing glucose, respectively (Figure 11D, left graphs). Some seeds from *briz2-3 abi5-7* plants that germinated on GM (+1% sucrose) had green cotyledons initially (Supplementary Figure S12A), but they did not expand fully, the green faded after several days, and by 10 days the seedlings were noticeably purple (Figure 11C, leftmost image and Supplementary Figure S12B).

In addition to inhibited germination on GM containing sucrose or glucose, *briz2-3 abi5-7* mutants had inhibited cotyledon greening and expansion (Figure 11). Ten days after plating, none of the *briz2-3 abi5-7* seedlings on GM plates containing sucrose or glucose had expanded green cotyledons, a significant difference compared to ~70% of the germinated seedlings on GM lacking sugar or on GM containing 1% mannitol in place of sucrose (58.3% of total seeds plated for both; Figure 11D, right graphs). We also noticed that seedlings grown on GM without sucrose from *briz2-3 abi5-7* plants, while green at 10 days, then yellowed/senesced earlier than seedlings from Col or *abi5-7* plants when maintained on the same plates for 2.5 weeks (Supplementary Figure S13). To summarize these results, sucrose inhibited germination of *briz2-3 abi57* mutants, while both sucrose and glucose inhibited cotyledon expansion and greening.

## Discussion

The two Arabidopsis *BRIZ* genes, *BRIZ1* and *BRIZ2*, were originally identified in a reverse genetic screen for phenotypic effects resulting from T-DNA insertion in genes encoding RING-type E3 ligases (Hsia and Callis, 2010). Although BRIZ1 and 2 share the same domains, single loss-of-functions mutants in either *briz1* or *briz2* have similar phenotypes, indicating that they are not functionally redundant. BRIZ1 and BRIZ2 interact *in vitro* and when over-expressed *in planta*, and expression of truncated BRIZ proteins unable to form heteromers fail to complement the *briz* mutant phenotypes, leading us to propose that the two proteins normally function in a complex as a single E3 ligase activity (Hsia and Callis, 2010). However, the biological functions of the BRIZ heteromer remained unknown.

We show here multiple lines of evidence to support the hypothesis that *briz1* and *2* embryos are hypersensitive to ABA. First, their macroscopic phenotypes resemble ABA-arrested seedlings: defective in germination, greening, true leaf formation and cotyledon expansion, while maintaining cell viability. *briz1* and *briz2* seeds are also defective in SCR, an ABA-modulated process. Although SCR was traditionally thought to be ABA-independent because SCR occurs on ABA-containing media that blocks subsequent radicle emergence, current evidence indicates that exogenous ABA does not penetrate through the seed coat or through the layer of cuticle outside the single cell layer of endosperm, which blocks ABA exposure to the embryo (De Giorgi et al., 2015). In nicking experiments, which expose embryos to the media, exogenous ABA is effective in blocking SCR in wild-type seeds (Barros-Galvão et al., 2019). In contrast, SCR in nicked *abi5-7* seeds is almost completely insensitive to exogenous ABA, indicating a key role for ABI5 in this process (Barros-Galvão et al., 2019). Next, the carotenoid biosynthesis inhibitor fluridone, which reduces endogenous ABA levels, partially relieved the growth arrest of *briz* embryos. This effect was reversed on media containing fluridone plus 0.1 μM ABA, a concentration that does not inhibit germination of wild-type seeds. The partial rescue of *briz2* growth in a genetic background of reduced ABA content and the partial rescue of both *briz* loci in the *abi5-7* mutant background additionally supports this model. Exogenous GA_3_, which functions antagonistically to ABA, promoted germination and cotyledon greening of *briz* mutants. Altogether, these results support the hypothesis that *briz* mutants are hypersensitive to ABA and that BRIZ plays a role in ABA signaling under non-stress conditions most predominantly during germination and early seedling growth.

Although *briz1* and *briz2* single mutants have the similar phenotypic differences from wild type, *briz1* mutants were not phenotypically rescued to the same extent as *briz2* mutants in either the *abi5-7* or *gin1-3* backgrounds. For example, while we were able to obtain mature *briz2-2 gin1-3* double mutant plants, we could not obtain mature *briz1-1 gin1-3* double mutants, likely as a result of increased double mutant seed loss observed in siliques of *BRIZ1/briz1-1 gin1-3* plants. These results suggest that the *in vivo* functions of BRIZ1 and BRIZ2 are not completely redundant. We previously observed that their *in vitro* biochemical activities were not equivalent (Hsia and Callis, 2010), and these differences could result in loss of *BRIZ1* having in a more severe effect *in vivo*, although we do not understand the mechanism.

The phenotype of *briz* mutants in the *abi5-7* background supports a model in which BRIZ is important during germination but is less important for later vegetative growth. Our hypothesis is that BRIZ acts as a brake on ABA signaling, and loss of BRIZ leads to ABA hypersensitivity in *briz* seeds. Loss of ABI5, which promotes inhibition of germination by ABA (Finkelstein and Lynch, 2000), partially compensates for the hypersensitivity to ABA in *briz abi5-7* double mutants and allows many seeds to germinate. However, their remaining ABA hypersensitivity is still strong enough to delay germination for an extended period of time. Once past germination and seedling establishment, the ability of *briz2-3 abi5-7* double mutants to grow to maturity with some seed set suggests that loss of BRIZ2 does not play a major role during later vegetative and reproductive growth. Whether BRIZ2 affects ABI5 accumulation, localization, and/or phosphorylation is challenging to assess. 72-h-old non-germinated *briz* seeds do have detectable ABI5 protein, but whether this results from the arrested germinated state or is causal in arresting growth is not certain.

How exactly might BRIZ function in ABA signaling? The roles of BRIZ orthologs could provide clues. Human BRAP2/IMP (BRCA1-associated protein 2, also called IMP for Impedes mitogenic signal propagation) is an E3 ligase with the same BRAP2, RING, ZnF UBP, and coiled-coil domains as *Arabidopsis* BRIZ1 and BRIZ2. BRAP2/IMP acts as a cytoplasmic retention factor for multiple proteins (Li et al., 1998; Asada et al., 2004; Davies et al., 2013) and reduces Ras/Raf/MEK/ERK signaling (Matheny et al., 2004). In Ras signaling, IMP sequesters the scaffold protein KSR1 and prevents KSR1 homo-oligomerization. In the presence of growth factor, IMP self-ubiquitinates and targets itself for proteasomal degradation. KSR1 is then free to homo-oligomerize and act as a scaffold for the kinase Raf and its substrate MEK. Like IMP, BRIZ could function as an adaptor protein between ABA signaling components.

Raf-like kinases and a MAPK scaffold protein have been identified in plants. There are 48 Raf-like kinases in *Arabidopsis* (Jonak et al., 2002), including CTR1 (also called *SIS1* for *SUGAR-INSENSITIVE1*). Though mostly known for its role in ethylene signaling, CTR1 also plays a role in sensitivity to both ABA (Ghassemian et al., 2000) and sugar (Zhou et al., 1998; Gibson et al., 2001). RAF10 and RAF11 are Arabidopsis Raf-like kinases that promote seed dormancy and ABA response (Lee et al., 2015). It was recently reported that multiple other Raf-like kinases are also involved in ABA signaling (Lin et al., 2020). RECEPTOR FOR ACTIVATED C PROTEIN KINASE 1 A (RACK1A) is an Arabidopsis MAPK scaffold protein (Guo et al., 2007; Cheng et al., 2015). Arabidopsis RACK1 proteins are encoded by three genes (*RACK1A, B, and C*; Chen et al., 2006). *rack1a* mutants are hypersensitive to ABA (Chen et al., 2006), and the increased ABA hypersensitivity of *rack1a rack1b* and *rack1a rack1c* double mutants suggests that the *RACK1* genes function redundantly to reduce ABA response (Guo et al., 2009). Whether BRIZ proteins have any relationship with Raf-like kinases or MAPK scaffold proteins is unknown. One hypothesis is that BRIZ might modulate the abundance or activity of a Raf-like kinase or a scaffold protein in an ABA-dependent manner.

We found that *briz1* and *briz2* embryos are hypersensitive to exogenous sucrose and glucose, which is not an osmotic effect. A number of ABA signaling mutants have altered sensitivities to sugar. For example, mutants that are insensitive to glucose include the PP2C phosphatase mutant *abi2-1* (Dekkers et al., 2008) and the transcription factor mutants *abi3-1* (Yuan and Wysocka-Diller, 2006; Dekkers et al., 2008), *abi4* (Arenas-Huertero et al., 2000), *abi5* (Arenas-Huertero et al., 2000), and *abf2* (Kim et al., 2004). Overexpression of ABI3, ABI4, or ABI5 results in glucose hypersensitivity (Finkelstein et al., 2002). Sugar-related phenotypes are evident in alternative names for many ABA-related genes. *ABA2* is also called *GIN1* for *GLUCOSE INSENSITIVE 1*, *ISI4* for *IMPAIRED SUCROSE INDUCTION 4*, and *SIS4* for *SUGAR INSENSITIVE 4*. *ABI3* is also called *SIS10* for *SUGAR INSENSITIVE 10*. *ABI4* is also called *GIN6* for *GLUCOSE INSENSITIVE 6* and has several other sucrose-based names including *SIS5* for *SUGAR INSENSITIVE 5*. With mutant phenotypes related to both to both ABA and sugar, *BRIZ* joins the collection of genes involved in both signaling pathways.

While *abi5-1* mutants are somewhat resistant to high levels of glucose (Arenas-Huertero et al., 2000), *briz abi5-7* double mutants retain sensitivity to glucose and sucrose. This indicates that *briz* sugar sensitivity occurs independent of ABI5. Curiously, *abi5-7 briz2-3* double mutants derived from homozygous *abi5-7 briz2-3* parents are more sensitive to sucrose than double mutants derived from heterozygous *BRIZ2/briz2-3* parents, and seeds from *abi5-7 briz2-3* plants are larger than seeds from *abi5-7 BRIZ2/briz2-3* plants. It is possible that in ABA-hypersensitive *briz2-3 abi5-7* plants, the transport of sugars or other nutrients to the developing embryo or seed is affected. The pigmentation visible in *briz* mutants plated on sucrose or glucose resembles descriptions of anthocyanins produced in response to high levels of exogenous sugar (Dekkers et al., 2008). The behavior of seeds from *briz2-3 abi5-7* mutants, which are hypersensitive to the 1% sucrose in agar GM plates compared to *briz2-3 abi5-7* mutants derived from *BRIZ2/briz2-3 abi5-7* plants, suggests that the parental *BRIZ2* genotype affects sugar sensitivity.

The reasons for heterogeneity in *briz* germination phenotypes, and for the partial rescue of some but not all *briz* seeds with reductions in ABA synthesis, ABA signaling or with the addition of GA_3_, are unknown. These variable responses could reflect a phenomenon called bet-hedging, where differences in germination behavior are observed even when genetically identical seeds are grown under the same environmental conditions. These differences can act as a mechanism to ensure species survival under possible variable, stressful conditions (Bradford, 2002; Mitchell et al., 2017; Cortijo and Locke, 2020). GA and ABA can act as a bistable switch to control germination, and this switch can amplify variability in germination (Abley et al., 2020). Perhaps *briz* individuals with altered ABA responses have amplified differences in ABA-GA antagonism during germination, with individuals “flipping” either one way or the other.

Gibberellic acid opposes most ABA responses by repressing ABA synthesis and ABA signaling, and the ratio of GA:ABA regulates the developmental outcome (Liu and Hou, 2018; Shu et al., 2018; Vishal and Kumar, 2018). The initial GA signaling event is GA-induced degradation/inactivation of the DELLA family of proteins. DELLAs suppress germination, in part by repressing expansin gene expression to prevent endosperm cell wall weakening and expansion (Sánchez-Montesino et al., 2019; Xu et al., 2020), and promote anthocyanin biosynthesis by sequestering the negative regulators JAZ and MYBL2, freeing other TFs to promote transcription of anthocyanin biosynthetic genes (Xie et al., 2016). Although it remains possible that defective GA signaling could contribute to the observed ABA hypersensitivity, possibly in an organ-specific and/or developmentally specific manner, we observed that *briz* seed germination is enhanced and seedling hypocotyls elongate in response to exogenous GA. Therefore, we are framing further experiments to test the hypothesis that the BRIZ ubiquitin E3 ligase plays a role in the ABA and sugar response network.

In summary, BRIZ1 and BRIZ2 are two RING-type E3 ligases involved in ABA signaling or response, either directly or indirectly. BRIZ is important for germination and early seedling growth, and further analysis of BRIZ function will provide insight into ABA’s role in regulating this critical developmental transition in the plant life cycle.

## Data Availability Statement

The raw data supporting the conclusions of this article will be made available by the authors, without undue reservation.

## Author Contributions

KL, MH, and JC designed the research and wrote the paper. KL, MH, Y-TC, and JC performed research and analyzed data. All authors contributed to the article and approved the submitted version.

### Conflict of Interest

The authors declare that the research was conducted in the absence of any commercial or financial relationships that could be construed as a potential conflict of interest.

## Footnotes

^1^https://abrc.osu.edu/

^2^https://www.danforthcenter.org/our-work/core-facilities/proteomics-mass-spectrometry/
