# Supplementary material for: The Arabidopsis thaliana E3 Ubiquitin Ligase BRIZ Functions in Abscisic Acid Response
Source: Front Plant Sci. 2021 Mar 16;12:641849. doi: 10.3389/fpls.2021.641849 (PMC8008127; doi:10.3389/fpls.2021.641849)
Supplement: Supplementary file 1 [file Data_Sheet_1.pdf]

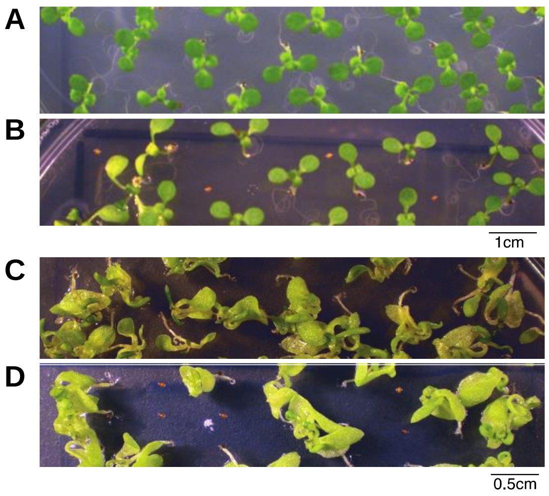

Figure S1. Addition of brassinolide does not affect the *briz* phenotype.

Seeds from *BRIZ/briz* plants on GM plates after 12 days of growth at 22°C. **(A)** Seeds from Col control on GM plate with DMSO (solvent control). **(B)** Seeds from a *BRIZ1/briz1-1* heterozygote on DMSO (solvent control). **(C)** Seeds from Col control on GM + 10 µM brassinolide. **(D)** Seeds from a *BRIZ1/briz1-1* heterozygote on GM + 10 µM brassinolide.

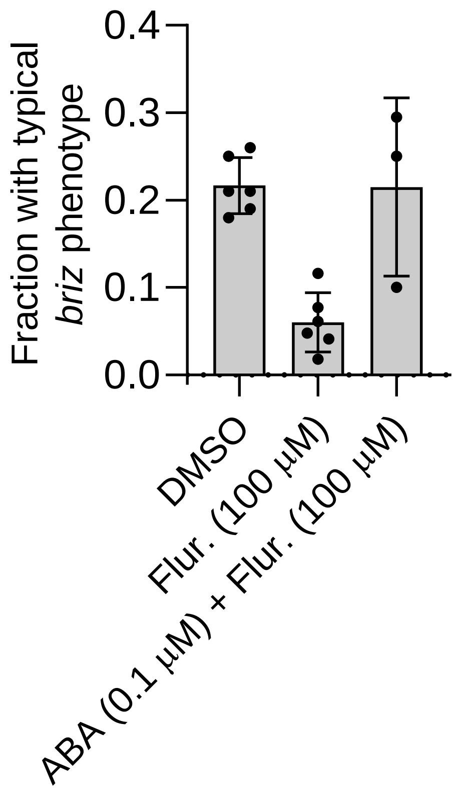

Figure S2. Fluridone reduces the fraction *of briz2-1* mutants with a typical *briz* phenotype, which returns upon addition of ABA.

Seeds from heterozygous *BRIZ2/briz2-1* plants were plated on agar GM plates containing 100 µM fluridone (Flur.) or 100 µM fluridone + 0.1 µM ABA. Plates with 0.1% DMSO were used as solvent control. The fraction of seedlings with a typical *briz* phenotype was scored after 12-15 days. Asterisks (**) represent $p < 0.01$ and Asterisks (***) represent $p < 0.001$ according to an ANOVA analysis (Graph Pad Prism).

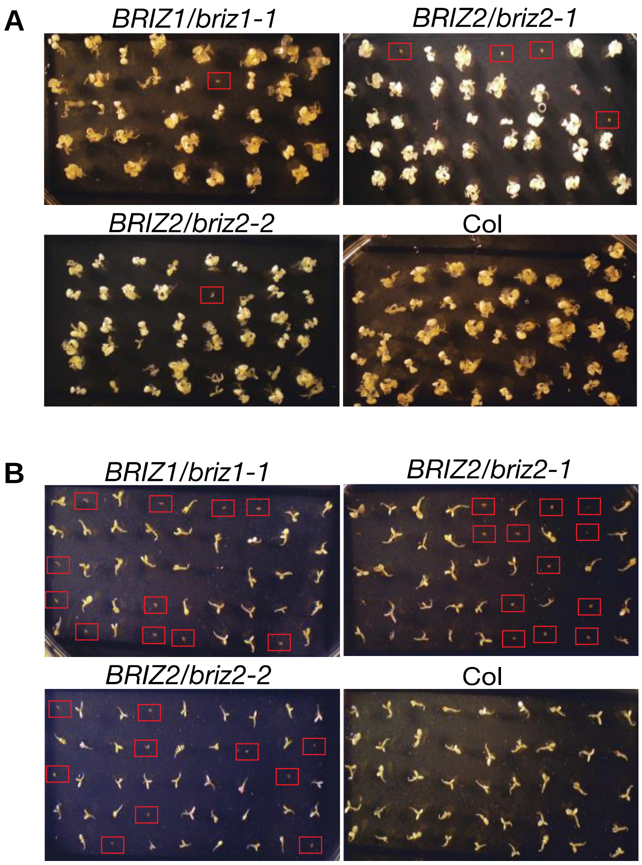

Figure S3. Phenotype of *BRIZ/briz* progeny on fluridone and fluridone + ABA plates.

(A) Top, progeny from *BRIZ1/briz1-1 and BRIZ2/briz2-1* and bottom, *BRIZ2/briz2-2 and* Col on GM plates with 100 µM fluridone. **(B)** As in (A), but on GM plates with 100 µM fluridone + 0.1 µM ABA. **(A, B)** Red boxes indicate individuals scored as *briz.* Bar represents 1 cm. Data are graphed with additional replicas in Figure 3 (for *briz1-1* and *briz2-2*) and in figure S2 (for *briz2-1*).

A

GM					GM + 0.1 µM ABA				
**Genotype**	**WT**	**briz**	χ^2	**P**	**Genotype**	**WT**	**briz**	χ^2	**P**
gin1-3	138	2	41.5	<0.0001	*gin1-3*	92	0	30.7	<0.0001
BRIZ2/briz2-2	35	12	0.007	=0.93	*BRIZ2/briz2-2*	36	13	0.06	=0.80
B2/briz2-2 gin1-3	203	21	29.2	<0.0001	*B2/briz2-2 gin1-3*	193	79	2.4	=0.12

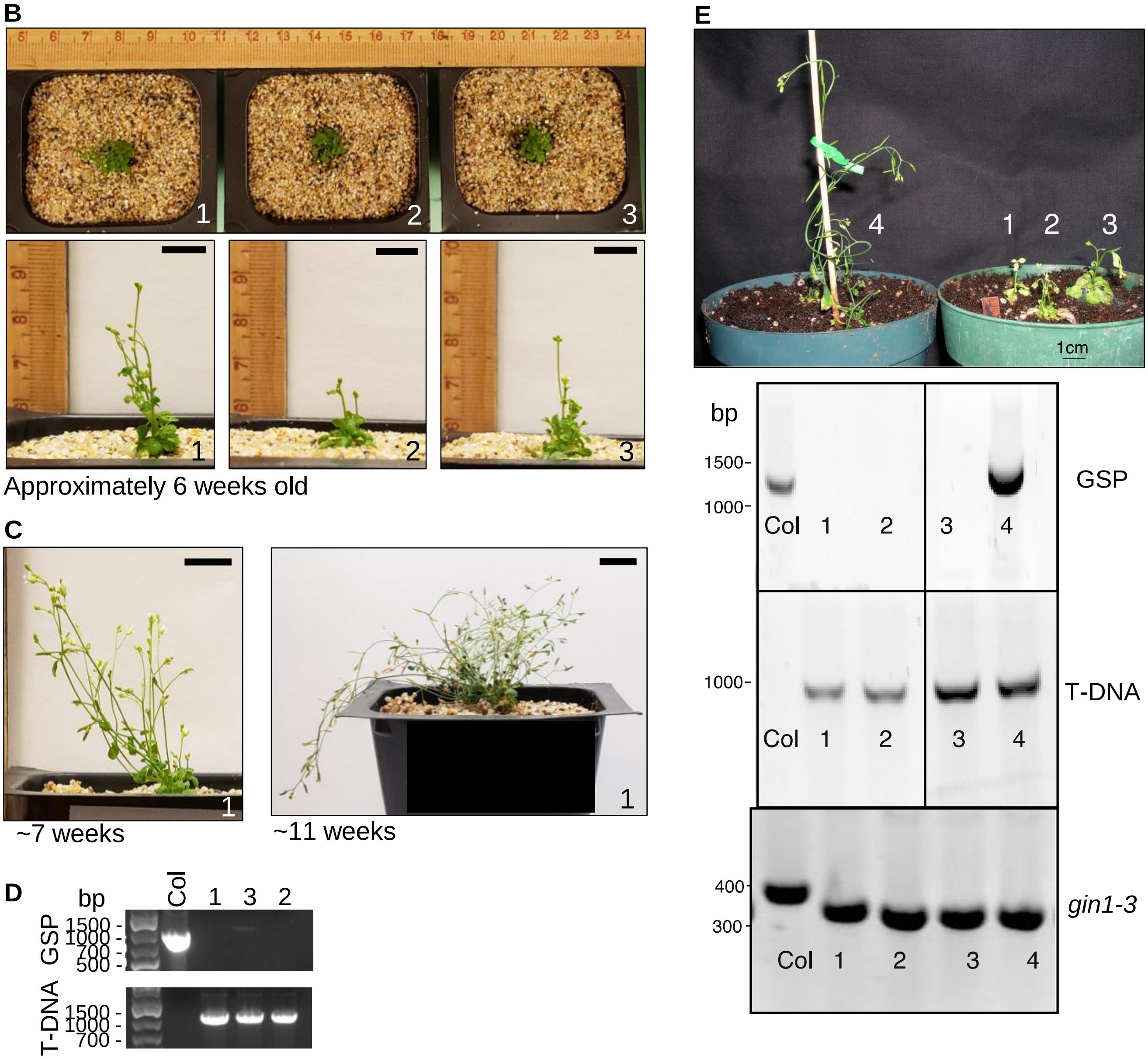

Figure S4. *briz2-2 gin1-3* double mutant analysis.

(A) Chi-square analysis of the *briz* phenotype in progeny from *gin1-3* single mutant, *BRIZ2/briz2-2*, and *BRIZ2/briz2-2 gin1-3* plants on GM media (left) or on GM + 0.1µM ABA. **(B)** *briz2-2 gin1-3* plants approximately 6 weeks after plating and transfer to pots with soil (sand on top). **(C)** Plant #1 at ~7 weeks (left) and ~11 weeks (right). **(D)** PCR genotyping of the three seedlings in (A) for *BRIZ2*. GSP, PCR using gene-specific primers flanking the T-DNA insertion site. T-DNA, PCR using a T-DNA primer and a flanking gene-specific primer. **(E)** Independent plating and growth of *briz2-2 gin1-3* double mutants (plants 1-3) compared to a *BRIZ2/briz2-2 gin1-3* sibling (4). Bottom, *briz2-2* (top two) and *gin1-3* (bottom gel) genotyping for plants above. *gin1-3* has a ~50 bp deletion compared to Col. Primers used for (D) and (E) are listed in Supplemental Table 1. Scale bars in plant panels represent 1 cm.

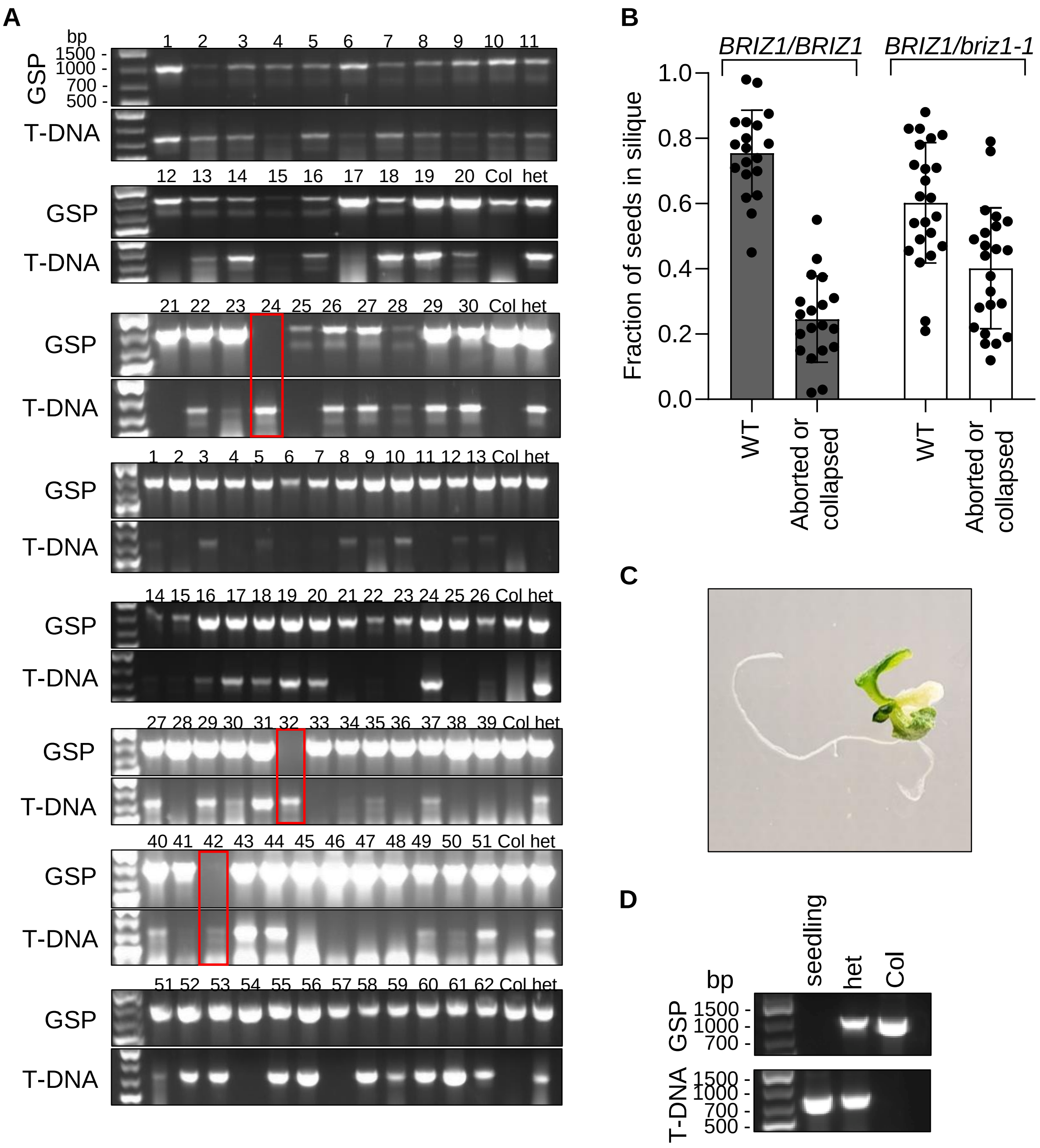

Figure S5. Most *briz1-1 gin1-3* double mutant seeds do not develop to maturity.

(A) 80 seedlings from a *BRIZ1/briz1-1* het in the *gin1-3* background were genotyped using PCR. Size markers for all gels are the same as for the top panel. Top 3 gels were used to genotype seedlings from one plate. Lower 5 gels were used to genotype seedlings from a second plate. Col and het are wild-type and het controls, respectively. Red boxes indicate possible *briz1-1* homozygotes. **(B)** Siliques of *gin1-3* plants that were wild-type or heterozygous for *BRIZ1* were examined. The phenotypes of seeds in each silique were recorded. Bars are SD. N = 19 WT siliques or 23 het siliques. **(C)** Photo of a homozygous *briz1-1* seedling, 26 days after plating. **(D)** PCR genotyping of the seedling in panel (C) for *BRIZ1*. GSP = PCR using gene-specific primers flanking the T-DNA insertion site. T-DNA = PCR using a T-DNA primer and a flanking gene-specific primer. Primers used are listed in Supplementary Table 1.

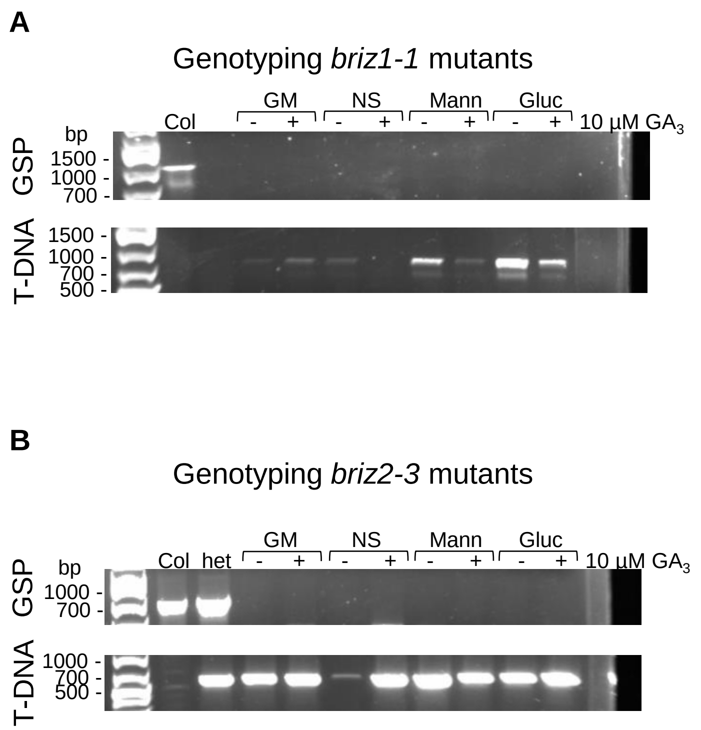

Figure S6. PCR genotyping of *briz1-1* and *briz2-3* single mutant excised embryos.

Genotypes of the **(A)** *briz 1-1* and **(B)** *briz2-3* embryos in Figure 7 were confirmed with PCR. GSP reactions use gene-specific primers flanking the T-DNA insertion site. T-DNA reactions use a T-DNA primer and a flanking gene-specific primer. Genotyping primers are listed in Table S1.

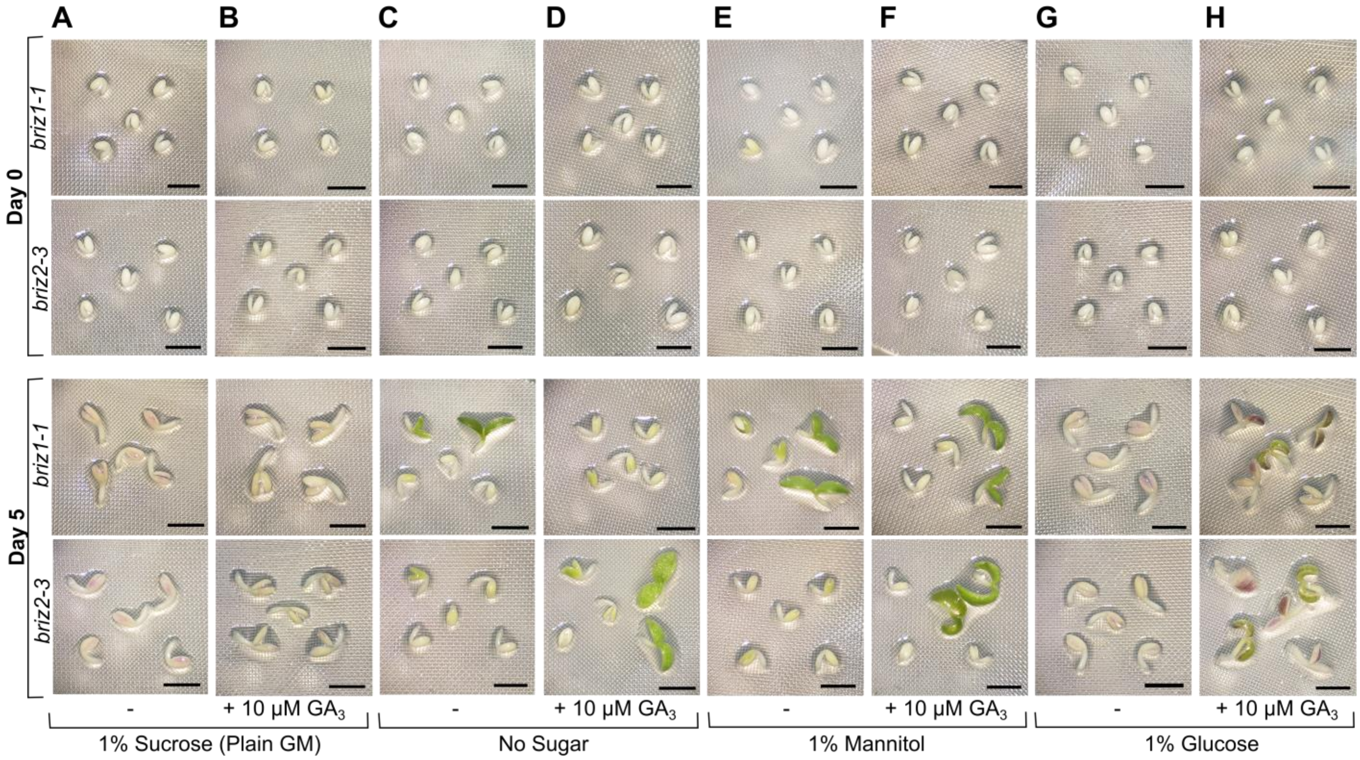

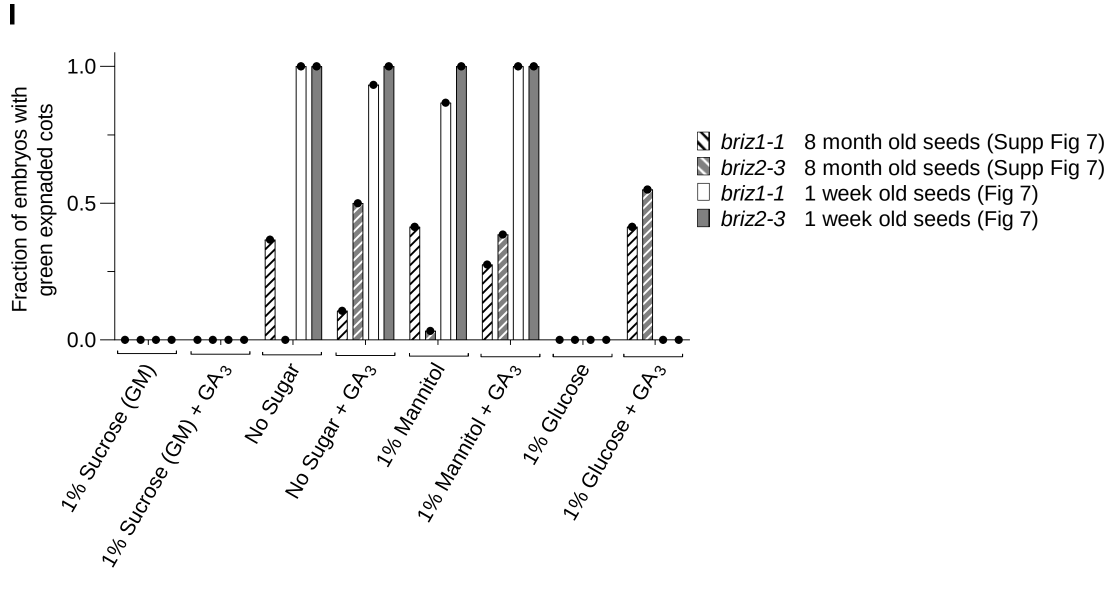

Figure S7. *briz1-1* and *briz2-3* embryos are hypersensitive to sucrose and glucose (using seeds dried for 8 months)

(A-H) Experiment was performed as described in Figure 7, but with seeds dried for approximately 8 months. **(I)** Comparison of cotyledon greening of embryos from seeds dried for 8 months (shown here in supplementary Figure 7), and embryos from seeds dried for 1 week (shown in Figure 7). For each type of growth media, the fraction of embryos with green expanded cotyledons was recorded and graphed. Striped bars indicate embryos from 8-month-old seeds, and solid bars indicate embryos from one week old seeds. N = 10 to 30 embryos per genotype on each type of growth media.

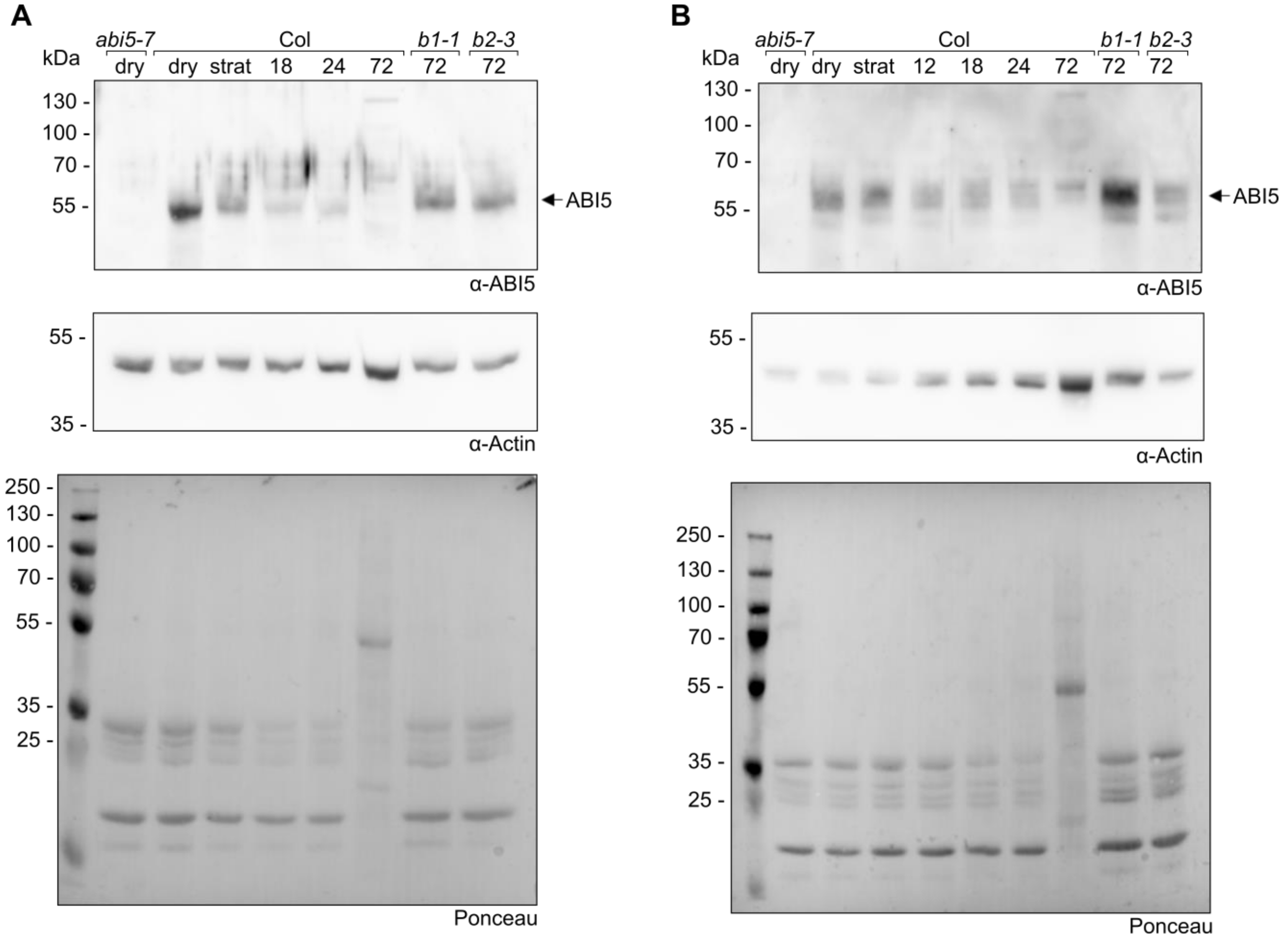

Figure S8. Additional replicates of experiment in Figure 8

(A) Second independent experiment, as described in Figure 8. **(B)** Third independent experiment, as described in Figure 8.

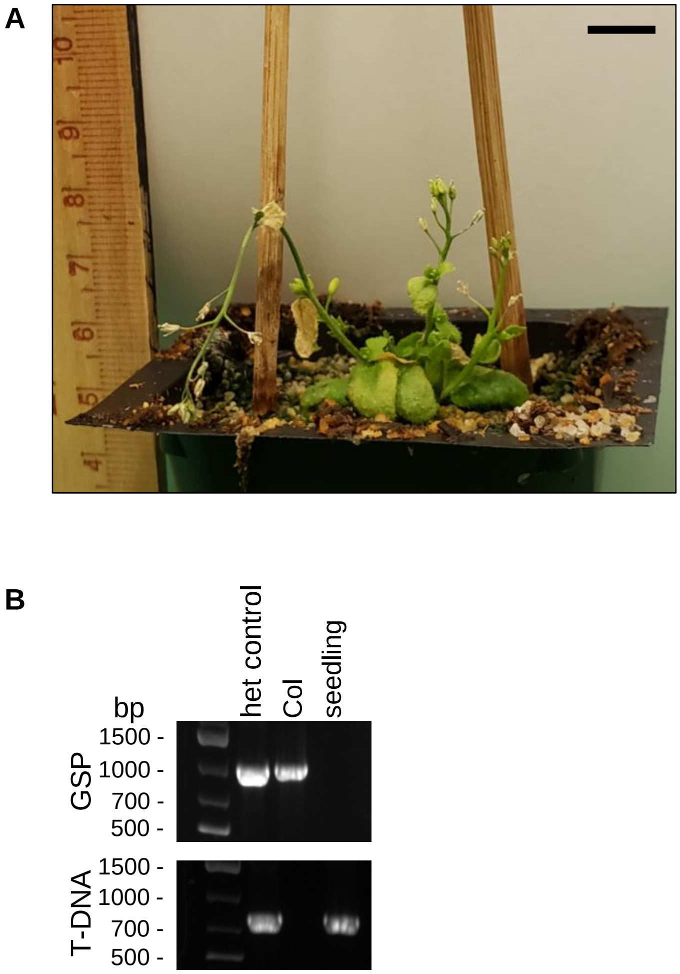

Figure S9. A *briz1-1 abi5-7* double mutant plant.

(A) Plant approximately 2 months after plating. Scale bar represents 1 cm. **(B)** PCR genotyping for *BRIZ1*. GSP = PCR using gene-specific primers flanking the T-DNA insertion site. T-DNA = PCR using a T-DNA primer and a flanking gene-specific primer. Primers used are listed in Supplementary Table 1.

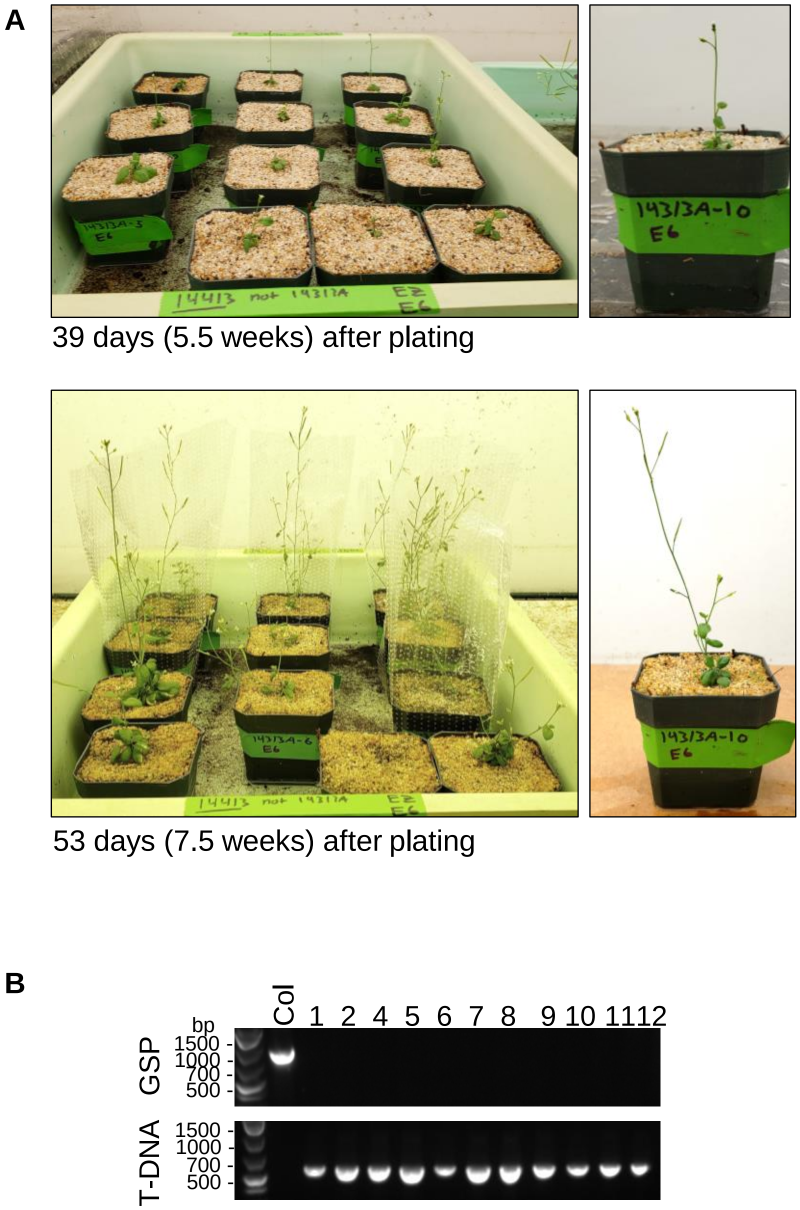

Figure S10. *briz2-3* mutants have additional growth in the *abi5-7* background.

(A) Out of 18 *briz2-3 abi5-7* plants transplanted to soil, 12 survived. Plants were photographed at 5.5 and 7.5 weeks. (These are not the plants pictured in Figure 5.5). **(B)** Eleven of the plants shown in panel A were genotyped for *BRIZ2* (plant #3 was too small to remove a leaf). GSP = PCR using gene-specific primers flanking the T-DNA insertion site. T-DNA = PCR using a T-DNA primer and a flanking gene-specific primer. Primers used are listed in Supplementary Table 1.

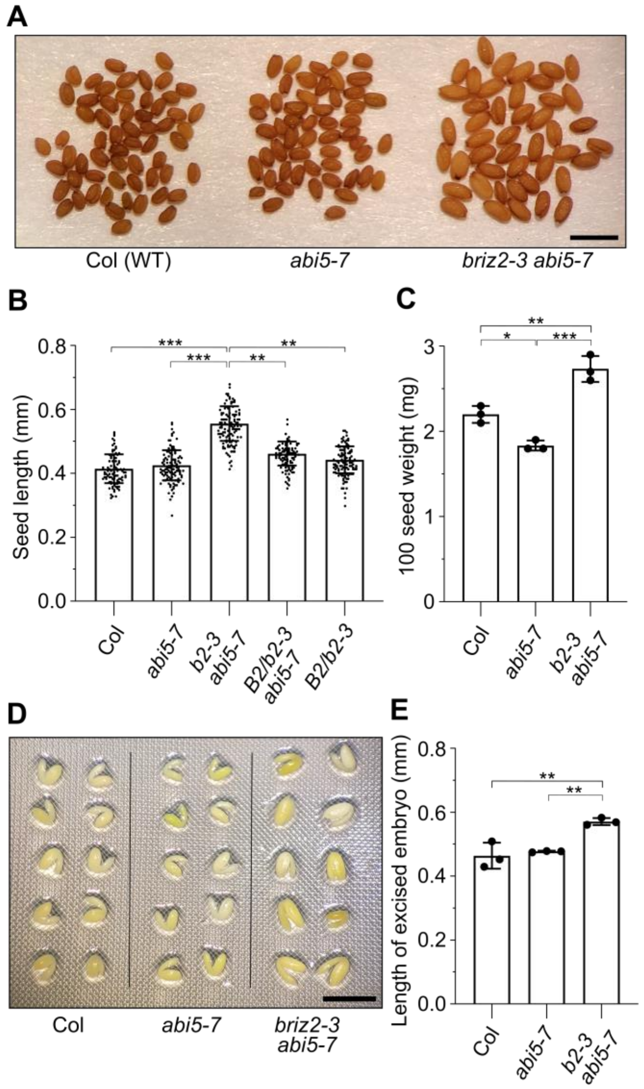

Figure S11. Seeds from *briz2-3 abi5-7* plants are larger and weigh more than WT or *abi5-7* seeds.

(A) Seeds from Col, *abi5-7*, and *briz2-3 abi5-7* plants. Scale bar represents 0.5 mm. **(B)** Lengths of seeds from Col, *abi5-7*, *briz2-3 abi5-7* double, *BRIZ2-3/briz2-3*, or *BRIZ2-3/briz2-3 abi5-7* plants were measured using ImageJ. N = two or three plants per genotype, with at least 50 seeds per plant. Each dot represents one seed. Bars represent SD. Asterisks (** or ***) represent a significant difference ($p < 0.01$ or $p < 0.001$, respectively) using ANOVA. **(C)** Weights of 100 seeds from Col, *abi5-7*, or *briz2-3 abi5-7* plants were measured. N = 3 groups of 100 seeds. Bars represent SD. Asterisks (*, **, or ***) represent significant differences with $p < 0.05$, $p < 0.01$, and $p < 0.001$, respectively, using ANOVA. **(D)** After 1 hour imbibition, embryos were excised from seed coats. Bar represents 0.5 mm. **(E)** Excised embryo length was measured using ImageJ. Bars represent SD. N = 3 groups of 10 embryos. Asterisks (**) represent a significant difference ($p < 0.01$) using ANOVA.

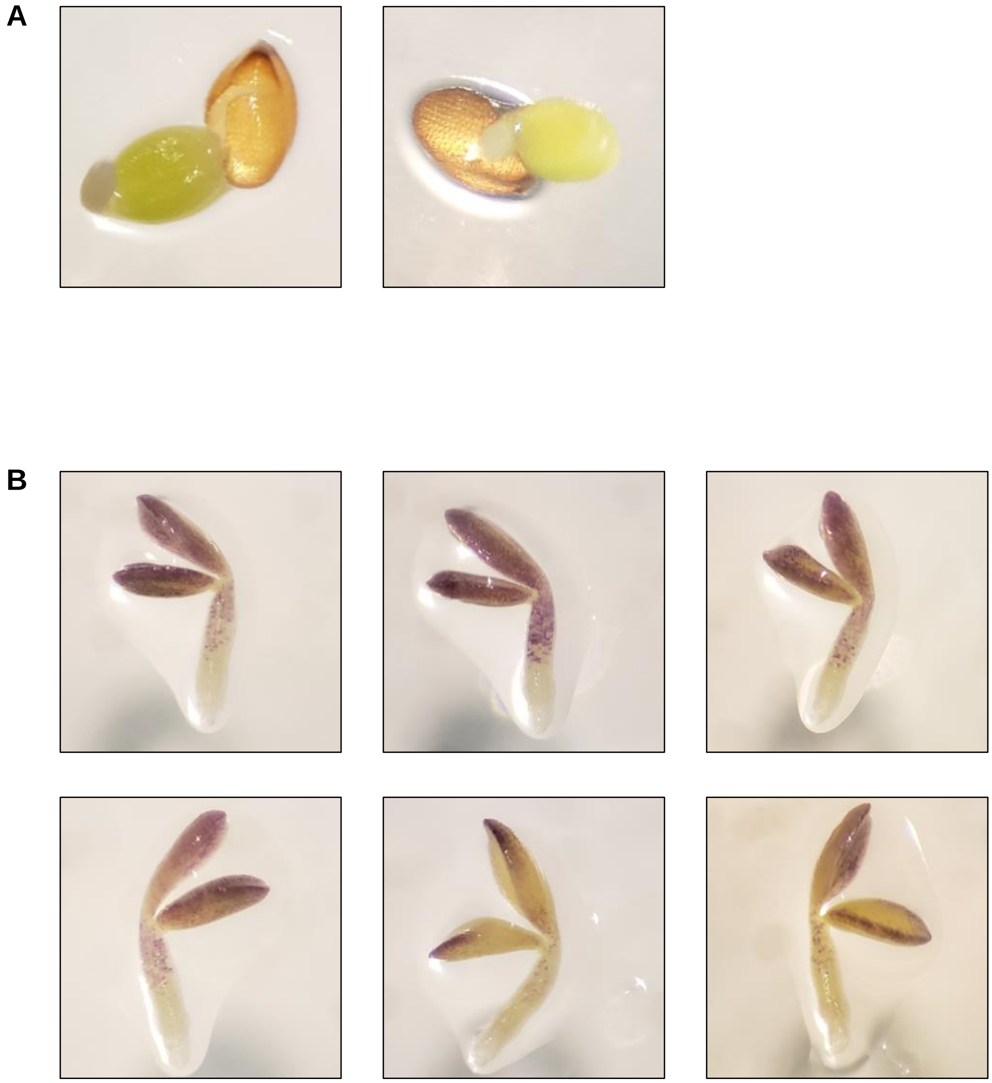

Figure S12. Many *briz2-3 abi5-7* embryos germinate with green cotyledons 1-3 weeks after plating but turn purple after several days and remain growth arrested.

briz2-3 abi5-7 seeds (F5 generation, collected 1 month before plating) were stratified at 4°C 3 for days, plated on agar GM plates, and grown under constant light. **(A)** Seeds started to germinate 1 week after plating (some took 2 or 3 weeks to germinate) and embryos had green cotyledons. Photos are of two different embryos. **(B)** Approximately three days after germinating, green faded and seedlings exhibited purple pigmentation. Photos are of six different seedlings (not the seedlings pictured in (A)).

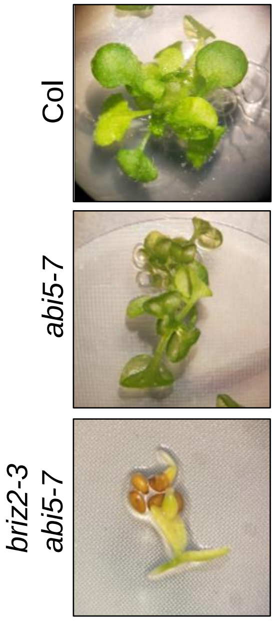

Figure S13. On GM lacking sucrose, F5 *briz2-3 abi5-7* double mutant seedlings fail to develop further and senescence earlier than *abi5-7* single or wild-type seedlings. Photos taken 2.5 weeks after plating.

Table S1 Primers for genotyping T-DNA lines.

Primer #	AGI Locus Code	Gene Name	Primer Sequence (5' to 3')	Description
**9-097**	At2g42160	*BRIZ1*	TCAAATGAATAGCACCTTAATGCAA	Forward primer for genotyping SALK_085207 (*briz1-1*). Use with 9-098.
**9-098**	At2g42160	*BRIZ1*	TTCAACCACCCTTAGCCATTTTT	Reverse primer for genotyping SALK_085207 (*briz1-1*). Use with 9-097 for gene specific, and with 9-001 for T-DNA junction.
**6-674**	At2g26000	*BRIZ2*	AGGCTAACAGGACAGGGTAGG	Forward primer for genotyping SALK_094761 (*briz2-1*). Use with 6-675.
**6-675**	At2g26000	*BRIZ2*	GCTTGCCAACTCTGTGCAG	Reverse primer for genotyping SALK_094761 (*briz2-1*). Use with 6-674 for gene specific, and with 9-001 for T-DNA junction.
**6-981**	At2g26000	*BRIZ2*	CACAACCATGTGCAATCATTC	Forward primer for genotyping SALK_151060 (*briz2-2*) and FLAG_122B09 (*briz2-3*). Use with 6-982.
**6-982**	At2g26000	*BRIZ2*	CTGCAGTCTCTGGCTTATTGC	Reverse primer for genotyping SALK_151060 (*briz2-2*) and FLAG_122B09 (*briz2-3*). Use with 6-981 for gene specific, with 9-001 for T-DNA junction for *briz2-2*, and with 8-133 for T-DNA junction for *briz2-3*.
**abi5-7 dCAPS-F**	At2g36270	*ABI5*	GTCGTCCATGGCGCAAGC	Forward primer for genotyping E74-1 (*abi5-7*). Use with abi5-7 dCAPS-R, then digest product with *Hin*fI. The *abi5-7* allele has two *Hin*fI sites.
**abi5-7 dCAPS-R**	At2g36270	*ABI5*	GATTGTTATTATTCTCCTCTGCGAT	Reverse primer for genotyping E74-1 (*abi5-7*). Use with abi5-7 dCAPS-F, then digest product with *Hin*fI. The *abi5-7* allele has two *Hin*fI sites.
**6-745**	At1g52340	*GIN1*	CCTCCTTTGCACGTTCTG	Forward primer for genotyping CS6147 (*gin1-3*). Use with 6-746. The *gin1-3* allele has a 50 bp deletion and will have a shorter PCR product.
**6-746**	At1g52340	*GIN1*	CTCTAACATCGCCATGGATG	Reverse primer for genotyping CS6147 (*gin1-3*). Use with 6-745. The *gin1-3* allele has a 50 bp deletion and will have a shorter PCR product.
**8-133**	-	-	CTACAAATTGCCTTTTCTTATCGAC	FLAG T-DNA left border primer
**9-001**	-	-	TGGTTCACGTAGTGGGCCATCG	SALK T-DNA left border primer
